# More Than Just a Periodontal Pathogen –the Research Progress on *Fusobacterium nucleatum*

**DOI:** 10.3389/fcimb.2022.815318

**Published:** 2022-02-03

**Authors:** Yuanxin Chen, Zhijie Huang, Zhengming Tang, Yisheng Huang, Mingshu Huang, Hongyu Liu, Dirk Ziebolz, Gerhard Schmalz, Bo Jia, Jianjiang Zhao

**Affiliations:** ^1^ Department of Oral Surgery, Stomatological Hospital, Southern Medical University, Guangzhou, China; ^2^ Department of Cariology, Endodontology and Periodontology, University of Leipzig, Leipzig, Germany; ^3^ Shenzhen Stomatological Hospital, Southern Medical University, Shenzhen, China

**Keywords:** *Fusobacterium nucleatum*, periodontal disease, halitosis, dental pulp infection, oral cancer, systemic diseases

## Abstract

*Fusobacterium nucleatum* is a common oral opportunistic bacterium that can cause different infections. In recent years, studies have shown that *F. nucleatum* is enriched in lesions in periodontal diseases, halitosis, dental pulp infection, oral cancer, and systemic diseases. Hence, it can promote the development and/or progression of these conditions. The current study aimed to assess research progress in the epidemiological evidence, possible pathogenic mechanisms, and treatment methods of *F. nucleatum* in oral and systemic diseases. Novel viewpoints obtained in recent studies can provide knowledge about the role of *F. nucleatum* in hosts and a basis for identifying new methods for the diagnosis and treatment of *F. nucleatum*-related diseases.

## 1 Introduction

*Fusobacterium nucleatum*, which exists in the oral cavity and gastrointestinal tract of humans, is an opportunistic pathogen causing different infectious diseases in the oropharynx and other parts of the oral cavity. These include appendicitis (Swidsinski et al., 2011), pericarditis (Truant et al., 1983), brain abscess (Han et al., 2003), osteomyelitis (Gregory et al., 2015), and chorioamnionitis (Altshuler and Hyde, 1988). *F. nucleatum* was first discovered in periodontal diseases and considered a potential periodontal pathogen (de Andrade et al., 2019). With improvements in microbial detection technology, a higher number of previously neglected microorganisms were found to play an important role in human diseases. Based on recent studies, *F. nucleatum* was associated with extra-oral malignancies, including colorectal cancer, breast cancer, esophageal squamous cell carcinoma, and gastric cancer (Kostic et al., 2012; Hsieh et al., 2018; Yamamura et al., 2019; Parhi et al., 2020). Moreover, the mechanisms of *F. nucleatum* affecting colorectal cancer (CRC) are important issues. Its role in extra-oral tumors suggests that it may also be important in oral cancer, and this has aroused a significant interest among scholars. However, its specific carcinogenic mechanisms in the oral field are unclear. Therefore, the role and specific mechanisms of this bacterium in different oral and extraoral diseases were examined.

**Figure 1:**
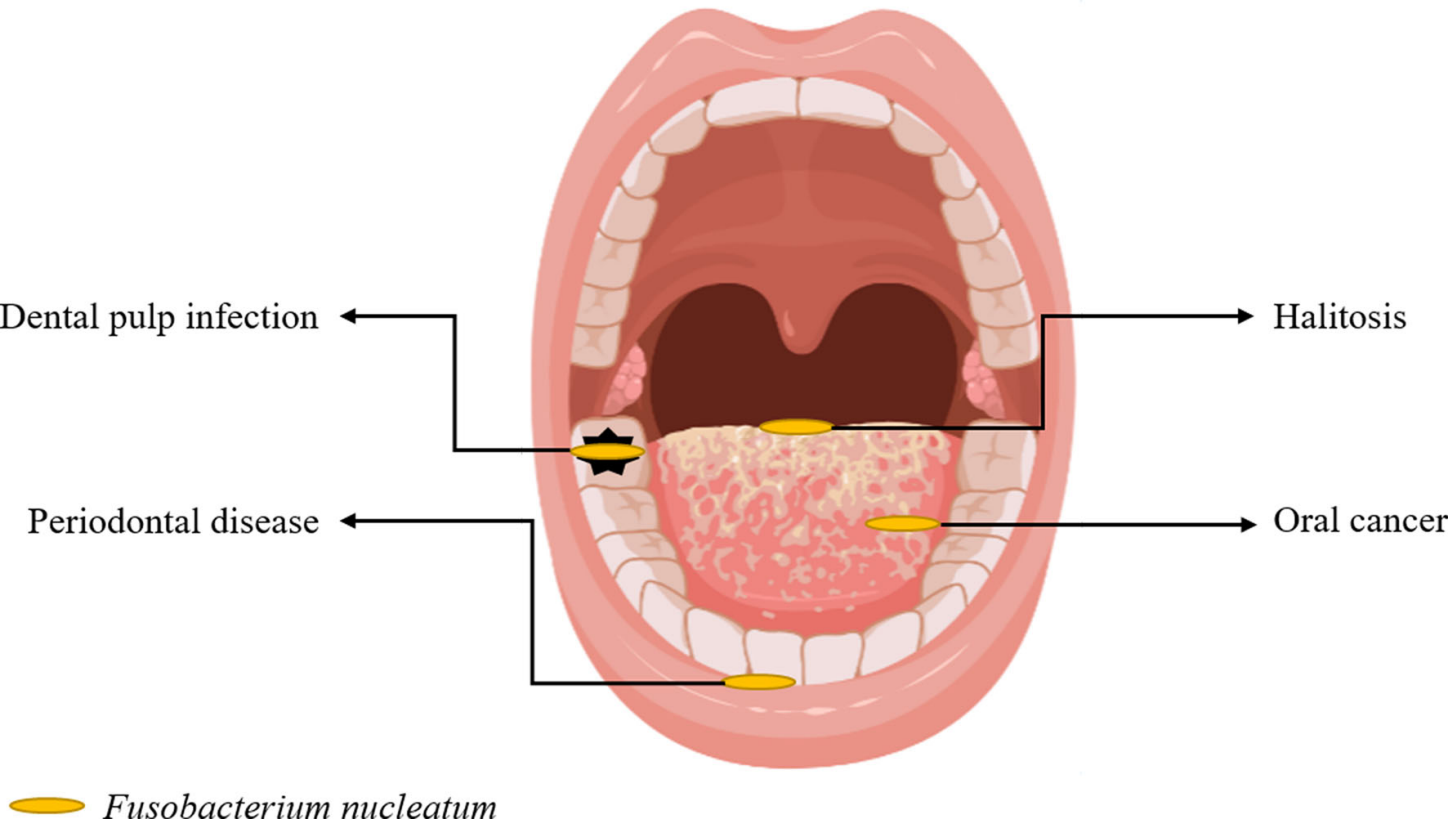
Oral Diseases Associated With *F. nucleatum*.

This narrative review focused on the role of *F. nucleatum* reported in the literature in recent years, which includes research progress in periodontal diseases, halitosis, dental pulp infection, oral cancer (**Figure 1**), and other related extraoral diseases.

## 2 *F. nucleatum*

*F. nucleatum* is an obligate anaerobic gram-negative bacillus belonging to the genus *Fusobacterium* and is named based on its slender shape and spindle-like tips at both ends (Bolstad et al., 1996). It exists in the human oral cavity, gastrointestinal tract, and other body parts. Moreover, it is a highly heterogenous species and is classified into five subspecies based on several phenotypic characteristics and DNA-DNA hybridization patterns, which are as follows: *F. nucleatum subsp. nucleatum*, *F. nucleatum subsp. polymorphum*, *F. nucleatum subsp. fusiforme*, *F. nucleatum subsp. vincentii*, and *F. nucleatum subsp. animalis* (Kim et al., 2010). In 2013, *F. nucleatum subsp. fusiforme* and *F. nucleatum subsp. vincentii* were classified into a single subspecies, *F. nucleatum subsp. fusiforme/vincentii* according to the phylogenetic analysis of 16S rRNA, rpoB, zinc protease, and 22 other housekeeping genes (Kook et al., 2013). Recently, the four *F. nucleatum* subspecies were classified into four *Fusobacterium* species (Kook et al., 2017). However, this notion has not been widely accepted in the fusobacterial community.

*F. nucleatum* can participate in the formation of dental plaques on human teeth and is correlated with the etiology of periodontitis (Patini et al., 2018). *F. nucleatum* is an important species in the physical interaction between gram-positive and gram-negative bacteria, which is a bridge between symbiotes and true pathogens planted on the surface of the teeth and epithelium (Kolenbrander, 2000). Adhesins which are found on the surface of *F. nucleatum* can attach to other bacteria and cells and contribute to bacterial pathogenicity. *Fusobacterium* adhesionA (FadA) is an adhesion protein and is the most significant virulence factor identified from *F. nucleatum* (Han, 2015). It exists as the intact pre-FadA, which comprises 129 amino acid residues, and the secreted mature FadA (mFadA), which comprises 111 amino acid residues (Xu et al., 2007). Pre-FadA and mFadA form FadAc, an active complex used for host cell binding and invasion (Xu et al., 2007; Témoin et al., 2012). FadA is highly conserved in oral *Fusobacterium*, such as *F. nucleatum* and *F. periodonticum*, but not in non-oral *Fusobacteria* (Han et al., 2005). Therefore, it can be a potential specific diagnostic marker for *F. nucleatum* and *F. periodonticum*.

The pathogenicity of *F. nucleatum* is mainly correlated with the following biological characteristics: First, several adhesins on its surface, including RadD, Aid1, and FomA, can co-aggregate bacteria to promote biofilm formation (Liu et al., 2010; Kaplan et al., 2014; Guo et al., 2017). Second, it can invade different host cells, such as epithelial cells, endothelial cells, and fibroblasts (Han, 2015). Third, it can produce different metabolites such as hydrogen sulfide, butyrate, and endotoxines released after cell death (Vital et al., 2014; Basic et al., 2017). Fourth, similar to other gram-negative bacteria, it can release extracellular vesicles or outer membrane vesicles (Liu et al., 2019; Liu et al., 2021), which contain several bioactive substances and participate in bacteria-bacteria or bacteria-host cells communications (Macia et al., 2019). Hence, due to the above-mentioned biological characteristics, *F. nucleatum* can be closely correlated with development of periodontal diseases, halitosis, dental pulp infection, oral cancer and extraoral diseases.

## 3 Association Between *F. nucleatum* and Periodontal Disease

Periodontal disease occurs in dental supporting tissues and comprises gingivitis and periodontitis. Dental plaque is the initiating and main pathogenic factor of periodontal disease. *F. nucleatum* is the dominant microorganism in periodontal tissues, and is associated with periodontitis etiology (Llama-Palacios et al., 2020).

### 3.1 Relevant Epidemiological Evidence

He et al. (2012) showed that periodontally healthy individuals and patients with chronic periodontitis commonly experience *F. nucleatum* infection, *F. nucleatum* is more abundant in patients with chronic periodontitis. Furthermore, the number of *F. nucleatum* in the subgingival plaque is significantly higher than that in the supragingival plaque and saliva. Wang et al. (2015) analyzed subgingival plaques in 29 healthy participants and 25 patients with chronic periodontitis *via* real-time polymerase chain reaction (PCR). Results showed that *F. nucleatum* could be detected in all patients with chronic periodontitis, and the detection rate in healthy participants was 86.21%. Moreover, it is more abundant in patients with chronic periodontitis than in healthy participants. The number of *F. nucleatum* increases with the severity of periodontal disease, progression of inflammation, and depth of periodontal pockets (Han, 2015). Rodrigues et al. (Arenas Rodrigues et al., 2018) performed culture and PCR of the subgingival biofilms of patients with gingivitis (n=70), periodontitis (n=75), and healthy individuals (n=95). The detection rates of *F. nucleatum* DNA were 57.1% and 68% in patients with gingivitis and periodontitis, respectively, and 37.8% in healthy individuals. Therefore, *F. nucleatum* may play a role in periodontitis progression.

### 3.2 Role of *F. nucleatum* in Periodontitis and Its Virulence Factors

*F. nucleatum* has a pathogenic role in periodontal infection. In an experimental periodontitis mouse model, *F. nucleatum* infection alone can cause alveolar bone loss or abscess (Chaushu et al., 2012). Co-infection caused by *F. nucleatum* and *Porphyromonas gingivalis* or *Tannerella forsythus* can stimulate host immune response and induce alveolar bone loss (Polak et al., 2009; Settem et al., 2012). The periodontal pathogenicity of *F. nucleatum* is correlated with its virulence factors, epithelial-mesenchymal transformation (EMT) of gingival epithelial cells, and the immune environment created at the lesion site.

#### 3.2.1 Virulence Factors

The virulence factors closely correlated with periodontitis in *F. nucleatum* include outer membrane proteins RadD, CmpA, Aid1, FomA, Fap2, and FadA; LPS; serine proteases; and butyric acid. The above-mentioned virulence factors can promote the development of periodontitis *via* different mechanisms.

In the process of dental plaque formation, *F. nucleatum* can co-aggregate with early and late dental plaque colonizers *via* related proteins and receptors in its outer membrane, thereby promoting the development of periodontal diseases (Kurgan et al., 2017). In particular, *F. nucleatum* can attach to early colonizers of dental plaques (including *Streptococcus*) *via* RadD (Guo et al., 2017), CmpA (Guo et al., 2017), and Aid1 (Lima et al., 2017) on its surface. After colonization in the biofilm, it can aggregate with the late colonizers of dental plaque (such as *P. gingivalis*) *via* FomA (Liu et al., 2010) and Fap2 (Coppenhagen-Glazer et al., 2015). *F. nucleatum* can gather together with the representatives of all oral bacterial species, thereby providing an important scaffold for the symplastic growth, development, and prosperity of these communities (Kabwe et al., 2019). Therefore, *F. nucleatum* can co-aggregate with periodontal pathogens in large quantities, thereby promoting the formation and maturation of dental plaque, the initiating factor of periodontal disease.

Previous studies have shown that the periodontal pathogenicity of *F. nucleatum* is correlated with its virulence factor FadA, which is not only an adhesin but also an invasive protein (Xu et al., 2007). By analyzing the whole genome of the genus *Fusobacterium*, (Umana et al., 2019) compared active invasive strains including *F. nucleatum* with passive invasive strains. Results showed that *F. nucleatum* invasion to cells was attributed to the synergistic action of FadA, RadD, and membrane occupation and recognition nexus protein (MORN2). Liu et al. (2014) revealed that the detection rate of the FadA gene of *F. nucleatum* was positively correlated with the gingival index. Hence, FadA may play an important role in periodontal diseases. FadA can bind to epithelial cadherin, invade host cells, and simultaneously affect the adhesion and connection between the cells. Hence, other microorganisms can invade the gingival epithelium. After invading epithelial cells, *F. nucleatum* can interact with intracellular receptor retinoic acidin-ducible gene I (RIG-I) *via* FadA to activate the nuclear factor kappa-B (NF-κB) pathway and then activate inflammatory response and can cause tissue destruction (Lee and Tan, 2014). Meng et al. (2021) showed that *F. nucleatum* can produce amyloid FadA under stress and disease, but not healthy, conditions. It can act as a scaffold for biofilm formation, endow acid tolerance and mediate the binding of *F. nucleatum* to host cells (Meng et al., 2021). In addition, amyloid FadA can induce periodontal bone loss in mice, and its toxicity can be weakened by amyloid binding compounds (Meng et al., 2021). Therefore, anti-amyloid therapies could be possible interventions for *F. nucleatum*-mediated disease processes.

If *F. nucleatum* dies and dissolves, it can release endotoxines, particularly lipopolysaccharide (LPS), which is recognized by Toll-like receptors on the surface of gingival epithelial cells and fibroblasts. Then intracellular danger signals are released, which activate the NLRP3 inflammasome to promote the release of mature cytokines such as interleukin-1β (IL-1β) (Hung et al., 2018), thereby enhancing periodontal inflammation and bone resorption. In addition, *F. nucleatum* can secrete a 65-kDa serine protease, which not only provides nutritional requirements for its growth but also destructs host tissues. By contrast, serine protease can degrade extracellular matrix proteins, leading to the destruction of periodontal connective tissues and immunoglobulins and complements in the host immune system. That is, it digests the α chain of IgA, which helps bacteria escape the host’s defense system (Bachrach et al., 2004). Butyric acid, which is another metabolite of *F. nucleatum*, may affect the destruction and healing of periodontal tissues. A high butyric acid concentration can promote the production of ROS in osteoblasts, thereby stimulating the secretion of 8-isoprostaglandin and matrix-metalloproteinase-2, which leads to bone destruction and affects bone repair (Chang et al., 2018).

#### 3.2.2 EMT

*F. nucleatum* can prompt the invasion of other periodontal pathogens by promoting the EMT of gingival epithelial cells. Abdulkareem et al. (2018) revealed that *F. nucleatum* and other gram-negative periodontal pathogens can promote the EMT of gingival epithelial cells, up-regulate the expression of Snail-1, down-regulate the expression of E-cadherin, and destruct the connection between epithelial cells. Then, the integrity of the gingival epithelium is lost, thereby promoting the invasion of pathogenic bacteria into the deep periodontal tissues.

#### 3.2.3 Immune Microenvironment

*F. nucleatum* can create a local immune microenvironment conducive for periodontal disease progression. Kurgan et al. (2017) found that both *F. nucleatum subsp. nucleatum* and *F. nucleatum subsp. polymorphum* can prevent the production of superoxide in neutrophils to prevent the oxidative killing of neutrophils. Simultaneously, all strains of *F. nucleatum* can reduce the number of neutrophils at the site of infection *via* both necrosis and apoptosis. Hence, *F. nucleatum* may promote the aggregation of late plaque colonizers such as *P. gingivalis* to the lesion by reducing the defensive function of neutrophils in the early stage of periodontal disease, thereby enhancing the development of periodontal diseases. Johnson et al. (2018) revealed that *F. nucleatum* alone can immediately trigger gingival inflammation, which is characterized by up-regulating the expression of IL-1β, IL-6, tumor necrosis factor necrosis factor-α (TNF-α), and HMGB1 and inducing macrophage infiltration in BALB/c mice. Simultaneously, infection contributes to the recruitment of osteoclasts. Meanwhile, IL-1 β and TNF- α can promote the development of osteoclasts (Boyce et al., 2005; Gao et al., 2007; Zupan et al., 2013). Therefore, the expression of pro-inflammatory cytokines increased by *F. nucleatum* infection is correlated with osteoclasts activation and further bone loss.

If *F. nucleatum* is recognized by Toll-like receptors TLR-2 and TLR-4, it activates the myeloid differentiation factor 88 dependent pathway. Hence, NF-κB is also activated, which may lead to the release of cytokines such as IL-6 and TNF-α (Kurgan et al., 2017; Kang et al., 2019). In addition, *F. nucleatum* can inhibit the proliferation of fibroblasts and promote their apoptosis, ROS generation and inflammatory cytokine production by activating the protein kinase B (PKB/AKT)/MAPK and NF-κB signaling pathways (Kang et al., 2019), thereby inhibiting tissue repair.

Moreover, *F. nucleatum* can work with other pathogenic bacteria to prevent immune system destruction. Compared with *F. nucleatum* infection alone, *F. nucleatum* and *P. gingivalis* co-infection in macrophages can passivate the activation of inflammasomes (Taxman et al., 2012). Moreover, *F. nucleatum* can increase the invasive potential of *P. gingivalis* (Saito et al., 2012). Hence, *F. nucleatum* can form an environment together with other pathogens that promotes inflammation and periodontal disease progression.

**Figure 2:**
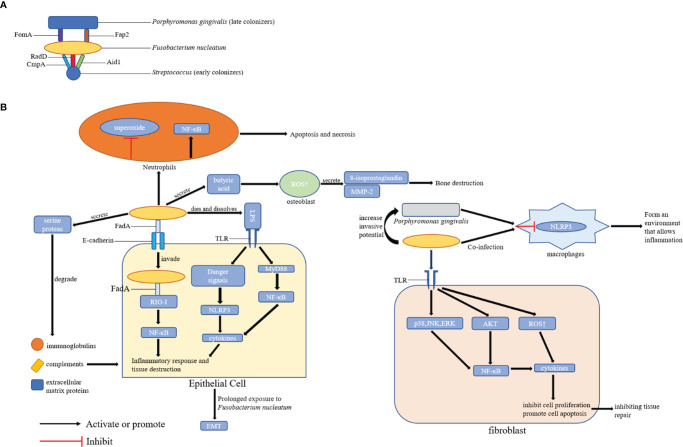
Possible Mechanisms of Periodontal Disease Promotion by *F. nucleatum*. **(A)** The role of *F. nucleatum* in the formation of dental plaque: *F. nucleatum* can coaggregate with early colonizers (including *Streptococcus*) and late colonizers (including *P. gingivalis*) of dental plaques *via* its adhesins; **(B)** The role of *F. nucleatum* in periodontal tissues: *F. nucleatum* can promote the development of periodontal disease by affecting epithelial cells, fibroblasts, and locally infiltrated neutrophils and macrophages in periodontal tissues. FomA, Fap2, CmpA, RadD, Aid1, and FadA are the major adhesins in *F. nucleatum*.

Although periodontitis is correlated with different microorganisms, *F. nucleatum* plays a key role in the development of periodontitis and the formation of dental biofilms. Moreover, it can interact with other pathogenic bacteria and create local inflammatory microenvironment, thereby accelerating periodontitis progression. However, the specific molecular mechanisms should be further evaluated. The above-mentioned mechanisms indicate that targeting *F. nucleatum* or its virulence factors may help enhance the effect of periodontal therapy and/or increase the efficacy of preventive strategies (**Figure 2**).

## 4 Association Between *F. nucleatum* and Halitosis

Halitosis is a common condition characterized by an unpleasant and disgusting odor emanating from the mouth (Hampelska et al., 2020). It can be classified into extra-oral halitosis and intra-oral halitosis. The latter is mainly caused by volatile sulfur compounds (VSCs). The most important VSCs are hydrogen sulfide, dimethyl sulfide and methyl mercaptane, which are mainly produced by anaerobic bacteria (Hampelska et al., 2020). In recent years, studies have found that halitosis is closely correlated with oral bacteria associated with periodontal disease on tongue coating, and *F. nucleatum* is enriched in the tongue coating of patients with halitosis.

### 4.1 Relevant Epidemiological Evidence

Amou et al. (2014) found that the tongue coating score was positively correlated with sensory value, methyl mercaptane concentration and VSC concentration in patients with bad breath. Meanwhile, the above-mentioned clinical indices of halitosis were positively correlated with the total number of oral bacteria and the abundance of *Prevotella intermedia*, *F. nucleatum* and *Campylobacter rectus*. In addition, the sensory value, VSC concentration and *Prevotella intermedia*, *F. nucleatum* and *Campylobacter rectus* concentrations of patients with bad breath who clean their tongue were significantly lower than those of patients without this habit (Amou et al., 2014). Adedapo et al. (2020) showed that *F. nucleatum*, *P. gingivalis* and *Prevotella intermedius* located on the back of the tongue are the main causes for the increased production of VSCs in patients with halitosis. Bernardi et al. (2019) revealed that the dorsal tongue biofilm of patients with halitosis had a significantly higher proportion of *F. nucleatum* and *Streptococcus*. In addition, in recent years, studies have revealed that psychological stress and anxiety can increase the discharge of VSCs in the oral cavity, which may be correlated with *F. nucleatum* (Nani et al., 2017; De Lima et al., 2020).

### 4.2 Treatment of *F. nucleatum*-Related Halitosis

In most cases, bad breath can improve with adequate moisture, proper dental care, oral hygiene, deep tongue cleaning, and, if necessary, garglinge with an effective mouthwash (Krespi et al., 2006). Since halitosis is closely associated with specific bacteria in the oral cavity including *F. nucleatum*, researchers have developed several treatments or related drugs for oral microbiota. These include vaccines, antibodies, plant extracts, chemical reagents, probiotics and photodynamic therapy to achieve the effect of treating or managing halitosis.

#### 4.2.1 Vaccines and Antibodies

Liu et al. (2010) revealed that the co-culture of *P. gingivalis* and *F. nucleatum* treated with FomA serum antibody reduced the production of VSCs. FomA is a porin on the surface of *F. nucleatum*, which has both adhesive and immunogenicity properties. Moreover, it participates in the co-aggregation of *F. nucleatum* and other oral microorganisms (Zhang et al., 2021). Therefore, FomA vaccine can inhibit halitosis by suppressing bacterial co-aggregation. In addition, mice immunized with the FomA vaccine can produce neutralizing antibodies and can effectively minimize the progression of gum abscesses caused by *F. nucleatum* and *P. gingivalis* co-infection (Liu et al., 2010). Moreover, an abscess in the gum pocket caused by bacterial infection is a common source of chronic halitosis (Liu et al., 2009). Compared with antimicrobial agents, vaccines are more selective to pathogenic bacteria and can prevent accidental injury of beneficial bacteria in the mouth due to the use of broad-spectrum antimicrobials, thereby resulting in potential adverse effects. Common antibacterial mouthwash can remove oral bacteria with nitrate reductase and affect the synthesis of nitric oxide *via* the nitrate–nitrite–nitric oxide pathway, resulting in higher blood pressure (Senkus and Crowe-White, 2020). The vaccine, which is more specific, can prevent similar problems.

In recent years, Wang et al. (2019) revealed that the use of egg yolk antibody IgY (obtained from the egg yolk of chickens stimulated by *F. nucleatum*) can inhibit the growth of *F. nucleatum* and significantly reduce the production of VSCs, volatile organic compounds and ammonia. Egg yolk antibody has a wide range of sources and is low cost. Considering the complexity of the halitosis mechanism, it is important to target specific pathogenic bacteria. Therefore, egg yolk antibody might become an ideal antibacterial agent for bad breath.

#### 4.2.2 Plant Extracts

Ben Lagha et al. (2020) found that three essential oils, specifically Labrador tea, peppermint and winter savory, could inhibit the growth and biofilm formation of *F. nucleatum* and reduce the production of VSCs by *F. nucleatum* in a dose-dependent manner. Therefore, these essential oils could not cause cytotoxicity to human oral keratinocytes in effective bactericidal concentration and action time. Higuchi et al. (2019) revealed that epigallocatechin gallate, the main component of green tea polyphenols, could inhibit the growth of *P. gingivalis*, *P. intermedia*, and *F. nucleatum* at a dose of 2.5mg/mL. Sun et al. (2019) showed that alkali-transformed saponin ATS-80 from quinoa husks separated by AB-2 resin has evident inhibitory effects against *F. nucleatum*. Further, its minimum inhibitory concentration at a dose of 31.3 μg/mL and minimum bactericidal concentration at a dose of 125 μg/mL are low. Hence, it can be used as an antibacterial agent for the treatment of halitosis. Xue et al. (2017) revealed that less polar ginsenosides obtained *via* thermal transformation have good antibacterial activity against *F. nucleatum*, *Clostridium perfringens*, and *P. gingivalis*. Ito et al. (2010) reported that myrsinoic acid B purified from Myrsine seguinii can inhibit the production of hydrogen sulfide and methyl mercaptane by *F. nucleatum*, *P. gingivalis*, and *Treponema denticola*. The above-mentioned plant extracts have a good inhibitory effect against oral bacteria that cause bad breath. However, due to their potential toxicity, oral microbiota dysbiosis and other side effects, their long-term application may require further evaluation.

#### 4.2.3 Chemical Reagents

Suzuki et al. (2018) found that Zn^2+^ ions can inhibit the growth of oral bacteria and the production of H_2_S, and its inhibition is strain-dependent, among which *F. nucleatum ATCC25586* is the most sensitive. Kang et al. (2017) revealed that both ZnCl_2_ and cetylpyridinium chloride (CPC) can effectively inhibit the growth of *F. nucleatum* and directly reduce the production of VSCs. *Via* randomized clinical trials, Shinada et al. (2010) revealed that gargling with ClO_2_ for 7 days could effectively reduce healthy subjects’ morning mouth odor, plaque, tongue coating, and the number of *F. nucleatum* in the saliva among healthy individuals. Similar to plant extracts, chemical reagents have a good bacteriostatic effect. However, they can also kill symbiotic bacteria in the oral cavity. Hence, this may lead to potential adverse effects and limit the possibility of their long-term application.

#### 4.2.4 Probiotics

Kang et al. (Shinada et al., 2010) isolated and identified three types of *Weissella cibaria* producing hydrogen peroxides from the saliva of children. These isolates can inhibit the production of VSCs by *F. nucleatum in vitro* and *in vivo*. Fujiwara et al. (2017) showed that Reuterin-related compounds can significantly inhibit methyl mercaptane produced by *F. nucleatum* and *P. gingivalis*. However, they have no cytotoxic effects on to human oral keratinocytes. Suzuki et al. (2014) found that *Lactobacillus saliva WB21* buccal tablets can significantly reduce the number of *F. nucleatum* in patients with oral odor. In the future, the use of probiotics can be a promising method to control bad breath, because they have not only antibacterial activity but also potential benefits to other systems of the whole body including the gastrointestinal tract.

#### 4.2.5 Photodynamic Therapy

Rai et al. (2016) found that the combination of photoactivated antibacterial methylene blue and 665nm laser can effectively kill *P. gingivalis*, *Prevotella intermedia*, *Peptostreptococcus anaerobius*, *Solobacterium moorei*, and *F. nucleatum*. Hence, photodynamic therapy may be a feasible method for the treatment of bad breath. Sigusch et al. (2010) showed that after periodontal scaling and root planning, patients with localized chronic periodontitis, in whom *F. nucleatum* could still be detected were treated with photodynamic therapy. Compared with the control group without photodynamic therapy, gingival redness and inflammation, bleeding on probing, average probing depth and clinical attachment level significantly decreased. Furthermore, the concentration of *F. nucleatum* DNA significantly reduced after 12 weeks of treatment. Antibacterial photodynamic therapy can effectively kill *F. nucleatum*, thereby indicating its potential role in the treatment of bad breath.

Considering that broad-spectrum antimicrobial agents can lead to microbiota dysbiosis, bacterial antibiotic resistance and other adverse consequences, researchers are committed to developing targeted drugs to kill *F. nucleatum* more accurately. These drugs include vaccines, antibodies, probiotics, and bacteriophages. However, most drug experiments are still in the *in vitro* test stage. Whether good antibacterial properties in the *in vitro* model can be achieved *in vivo* remains unknown, and the effective dose and safety must be further evaluated.

## 5 Association Between *F. nucleatum* and Pulp Infection

At present, *via* the detection of bacteria in dental pulp infection samples, several studies have found that *F. nucleatum* is significantly abundant in respective samples. Thus, it may play an important role in the development and progression of dental pulp infection.

### 5.1 Relevant Epidemiological Evidence

In pulpitis samples, Sassone et al. (2008) used checkerboard DNA–DNA hybridization to determine the composition of the microbiota of primary pulp infection. Several species such as *F. nucleatum ssp. vincentii*, *Veillonella parvula*, *Treponema socranskii*, *Enterococcus faecalis*, and *Campylobacter gracilis* found in symptomatic cases and *F. nucleatum ssp. vincentii*, *F. nucleatum ssp. nucleatum*, *Enterococcus faecalis*, *Eubacterium saburreum*, and *Neisseria mucosa* in asymptomatic cases. In the samples of periapical periodontitis, Bouillaguet et al. (2018) analyzed the bacteria in the dentin and root canal samples of teeth with primary periapical periodontitis and secondary apical periodontitis *via* 16S rRNA gene amplification sequencing. Results showed that *F. nucleatum* is the most common and abundant operational taxonomic unit. Meanwhile, the proportion of *F. nucleatum* in secondary root canal infection was lower than that in primary root canal infection. Rôças et al. (Rôças and Siqueira, 2012) showed that the most common taxa detected in the microbiota of retreated root canals were *Propionibacterium species*, *F. nucleatum*, *streptococci*, and *Pseudoramibacter alactolyticus*. Pereira et al. (2017) quantitatively detected bacteria in apical 3 mm and periapical infection samples of teeth with failed pulp treatment. Results showed that *F. nucleatum*, *Dialister pneumosintes*, and *Tannerella forsythia* were the most common bacteria. Barbosa-Ribeiro et al. (2021) detected the microbiota in the root canal of teeth with failed pulp treatment *via* 16S rRNA gene sequencing and PCR. Results showed that *Enterococcus faecalis*, *F. nucleatum*, and *P. gingivalis* were associated with periapical lesions measuring > 3 mm. Johnson et al. (2006) found that *Enterococcus faecalis* and *F. nucleatum* can co-aggregate. Hence, the combination of these two bacteria plays a potential role in dental pulp infection. In addition to evidence obtained using samples of pulpitis and periapical periodontitis, *F. nucleatum* was found to be abundant in endo-periodontal lesions. Didilescu et al. (2012) qualitatively and semi-quantitatively evaluated the bacteria in the root canal system and periodontal pocket of 46 patients with endo-periodontal lesions *via* PCR and DNA–DNA blotting. *Parvimonas micra*, *F. nucleatum*, and *Capnocytophaga sputigena* were extremely abundant in dental pulp samples, thereby showing that these bacteria may play a role in the pathogenesis of endo-periodontal lesions. Based on the evidence obtained using the above–mentioned clinical samples, *F. nucleatum* may play a role in dental pulp infection and disease progression (**Table 1**).

**Table 1:** Alterations in Predominant Bacteria Identified in Dental Pulp Infection (Sassone et al., 2008; Didilescu et al., 2012; Rôças and Siqueira, 2012; Pereira et al., 2017; Bouillaguet et al., 2018; Barbosa-Ribeiro et al., 2021).

Bacterial Phylum/Genus/Species	Samples	Testing methods
In symptomatic cases: *F. nucleatum subsp. Vincentii, Veillonella parvula, Treponema socranskii, Enterococcus faecalis, and Campylobacter gracilis*	30 Symptomatic and 30 asymptomatic single-rooted teeth with necrotic pulp	Checkerboard DNA-DNA hybridization method
In asymptomatic cases: *F. nucleatum subsp. Vincentii, F. nucleatum subsp. nucleatum, E. faecalis, Eubacterium saburreum, and Neisseria mucosa*		
The most prevalent and abundant OUT: *F. nucleatum*	43 dental roots(21 primary apical periodontitis group and 22 secondary apical periodontitis group) and 21 dentin samples	16S rRNA gene sequencing
The proportions of *F. nucleatum*: higher in primary infected root canals and lower in secondary infected root canals		
The most prevalent taxa: *Propionibacterium species, F. nucleatum, streptococci, and Pseudoramibacter alactolyticus*	42 teeth undergoing root canal retreatment	Quantitative real-time PCR (qPCR) assay
The most prevalent species: *F. nucleatum (71.6%), Dialister pneumosintes (58.3%) and Tannerella forsythia (48.3%)*	33 3 mm samples root ends and 30 samples of the surrounding chronic periapical infection	Quantitative real-time PCR (qPCR) assay
The most prevalent species: *E. faecalis and Porphyromonas gingivalis*	20 infected root canals of single-rooted teeth at the different phases of the endodontic retreatment	16S rRNA gene sequencing and PCR
Endodontic samples: *P. micra, F. nucleatum and C. sputigena*	46 patients presenting with different types of endo-periodontal lesions	PCR and DNA–DNA hybridization
Periodontal samples: *P. micra, F. nucleatum, C. sputigenaplus and C. rectus*		

### 5.2 Possible Mechanisms of *F. nucleatum* in Dental Pulp Infection

The specific role and mechanisms of F. nucleatum in dental pulp infection remain unclear. Current studies have shown that the role of F. nucleatum in dental pulp infection may be correlated with its alkali tolerance and endotoxines.

#### 5.2.1 Alkali Tolerance

Lew et al. (2015) found that *F. nucleatum* could tolerate the root canal environment with pH 9, and the alkali tolerance of *F. nucleatum* in the biofilm was stronger than that of *F. nucleatum* in the planktonic state (Chávez de Paz et al., 2007). It was concluded that its strong alkali tolerance made it survive in the root canal washed by alkaline disinfectant, allowing its existence in all stages of dental pulp infection.

#### 5.2.2 Endotoxines

Endotoxines on the surface of *F. nucleatum* may play an important role in root canal infection. Gomes et al. (2012) reported that in the teeth with primary dental pulp infection, the root canals with clinical symptoms had higher endotoxin content than asymptomatic teeth, and there was a positive correlation between endotoxin content and larger X-ray permeable areas (> 3 mm) (Barbosa-Ribeiro et al., 2021). *Enterococcus faecalis*, *F. nucleatum*, and *P. gingivalis* are associated with periapical lesions measuring > 3 mm. Thus, the endotoxines of pathogenic bacteria may play a role in the progression of root canal infection. Martinho et al. (2014) found that *P. micra*, *F. nucleatum*, and *P. gingivalis* were the most common bacteria in infected root canals. Meanwhile, endotoxines in the root canals were positively correlated with IL-6 and IL-10. After macrophage stimulation due to the contents of infected root canals, the phosphorylation of p38 reached the peak at 60 min, and NF-kB was activated rapidly 10 min after stimulation. Hence, the pathogens in the root canal may activate the TLR-4 of macrophages *via* endotoxines and can promote the production of IL-6 and IL-10 *via* the p38 MAPK and NF-kB signaling pathways, thereby leading to root canal inflammation. Maciel et al. (2012) inoculated *F. nucleatum* into the root canals of sterile mice. Then, *F. nucleatum* upregulated the expression of IFN-γ and TNF-α mRNA in periapical tissues on the 7th (acute phase) and 14th (chronic phase) days of infection. Notably, IFN-γ may interact with TNF-α, induce RANKL overexpression, and activate osteoclast bone resorption (Fukada et al., 2009; Teixeira-Salum et al., 2010). Under bacterial stimulation, RANKL and pro-inflammatory cytokines induce a synergistic effect in the periapical area, thereby promoting the expansion of periapical lesions (Kawashima et al., 2007; De Rossi et al., 2008).

Based on the above-mentioned pathogenic mechanisms of F. nucleatum, the use of conventional alkaline disinfectants may not be effective in refractory dental pulp infections. After relevant pathogenic bacteria are identified *via* bacterial culture, better outcomes can be achieved using targeted drugs for killing bacteria.

## 6 Association Between *F. nucleatum* and Oral Cancer

Oral cancer is the 11th most common cancer worldwide, and oral malignant tumors are oral squamous cell carcinoma (OSCC) accounts for approximately 90% of all oral malignancies (D’Souza and Addepalli, 2018). Surgical techniques, adjuvant radiotherapy and chemotherapy have progressed in recent decades. However, the incidence of OSCC may increase worldwide, and the 5-year overall survival rate is extremely low at approximately 50%–60% (Zhang et al., 2019). According to the literature, approximately 15% of OSCC cases have an unknown origin and can be attributed to viruses (such as *human papilloma virus* and *Epstein*–*Barr virus*), fungi (such as *Candida albicans*) and certain bacteria. Bacterial infection can lead to chronic inflammation, and chronic inflammation caused by infection is one of the most important causes of cancer (Kuper et al., 2000). Therefore, in some cases of OSCC of unknown origin, the biological role and related mechanisms of specific microorganisms, which have scientific significance and clinical application value for the prevention, early diagnosis and treatment of OSCC, should be further explored.

### 6.1 Relevant Epidemiological Evidence

*F. nucleatum*, a common opportunistic bacterium in the oral cavity, is closely correlated with oral cancer in recent years. Yang et al. (2018) performed 16S rRNA V3V4 amplification sequencing to determine the microbiota in the mouthwashes of 51 healthy people and 197 patients with OSCC at different stages. Results showed that the abundance of *Fusobacteria* increased significantly with oral cancer progression among healthy controls (2.98%) those with OSCC stage 1 (4.35%) to 4 (7.92%). Meanwhile, at the genus level, the abundance of *Fusobacterium* increased with cancer progression. *Via* 16S rRNA amplification sequence of oral swabs, Su et al. (2021) analyzed the bacteria within the lesion surface of OSCC and its contralateral normal tissues of male patients with buccal mucosal cancer in the cohort of discovery (n = 74) and subsequent validation cohort (n = 42). Hence, the bacterial biomarkers were associated with OSCC, among which the most different genera were *Fusobacterium* (enriched in OSCC) and *Streptococcus* (reduced in OSCC). Further functional prediction of the oral microbiome showed that there was a differential enrichment of microbial genes correlated with terpenoid and polyketide metabolism between the control and tumor groups. Hence, oral microbiome played a role in the formation of the tumor microenvironment by inhibiting the biosynthesis of secondary metabolites with anticancer effect (Su et al., 2021). At the species level, Chang et al. (2019) detected the relative abundance of *P. gingivalis*, *F. nucleatum*, and *Streptococcus sanguis* in 61 cancer tissues, paracancerous tissues, subgingival plaque samples and 30 normal tissues *via* quantitative polymerase chain reaction (qPCR). The numbers of *P. gingivalis* and *F. nucleatum* in cancer tissues were higher than those in normal and paracancerous tissues. Moreover, the number of *Streptococcus sanguis* in normal tissues was higher than that in malignant and paracancerous tissues. In addition, the relative abundance of *P. gingivalis* and *F. nucleatum* in cancer tissues was positively correlated with their relative abundance in subgingival plaque. Al-Hebshi et al. (2017) sequenced the V1-V3 DNA of 20 fresh OSCC biopsy and 20 deep epithelial swab samples. Results showed that *F. nucleatum subsp. polymorphum* had the highest proportion in oral cancers, followed by *Pseudomonas aeruginosa* and *Campylobacter*. Zhang et al. (2019) performed 16S rDNA sequencing to analyze the microbiota compositions of tumor sites and opposite normal tissues in the buccal mucosal of 50 patients with OSCC. Results showed that the richness and diversity of bacteria were significantly higher in tumor sites than in controls. The abundance of *F. nucleatum*, *Prevotella intermedia*, *Aggregatibacter segnis*, *Capnocytophaga leadbetteri*, and *Peptostreptococcus stomatis* increased significantly. In the above-mentioned clinical samples, *F. nucleatum* was enriched in OSCC. Therefore, it may have a certain effect on the development of OSCC (**Table 2**).

**Table 2:** Alterations in Predominant Bacteria Identified in Oral Cancer (Nagy et al., 1998; Al-Hebshi et al., 2017; Yang et al., 2018; Yost et al., 2018; Chang et al., 2019; Zhang et al., 2019; Hosgood et al., 2021; Su et al., 2021).

Bacterial Phylum/Genus/Species	Subjects	Types of Samples	Testing methods	Association with Oral Cancer
*Fusobacterium*	20 male and 1 female	Biofilm from the central surface of the lesions and from contiguous healthy mucosa	ATB identification procedures (BioMerieux, Lyon, France)	Increased at tumor sites
*Fusobacterium*	40 Chinese subjects	Cancer lesion samples and matched controls	16S rRNA gene sequencing	Significantly enriched in OSCC samples; several operational taxonomic units (OTUs) associated with *Fusobacterium* were highly involved in OSCC and demonstrated good diagnostic power
*Fusobacteria*	4 OSCC subjects and 7 healthy subjects	Oral swab	Metatranscriptomic analysis	*Fusobacteria* virulence factors may be involved in the pathogenesis of oral cancer
*Fusobacterium*	6 patients with OSCC	The Cancer tissues, paracancerous tissues and subgingival plaque samples	16S rRNA gene sequencing	Had significantly higher relative abundances in cancer tissues than in paracancerous tissues
*Fusobacterium*	51 healthy individuals and 197 OSCC patients	Oral rinse from 51 healthy individuals and 197 OSCC patients at different stages	16S rRNA gene sequencing	Increased with cancer progression
*Fusobacterium*	Discovery (n=74) and validation (n=42) cohorts of male patients with cancers of the buccal mucosa.	Buccal swab	16S rRNA gene sequencing	Enriched in the tumor sites
*F. nucleatum subsp. polymorphum*	20 OSCC patients and 20 control subjects	Fresh biopsies (cases) and deep-epithelium swabs (matched control subjects)	16S rRNA gene sequencing	The most significantly overrepresented species in the tumors
*F. nucleatum*	50 patients with OSCC	Tumor sites and opposite normal tissues in buccal mucosal	16S rDNA sequencing	Significantly increased in the OSCC group

### 6.2 Possible Role and Related Mechanisms of *F. nucleatum* in Oral Cancer

The enrichment of *F. nucleatum* in OSCC has attracted the attention of scholars. However, its role in OSCC and specific mechanisms are not completely elucidated. Zhang et al. (2021) analyzed the differentially expressed mRNAs and lncRNAs caused by human immortalized oral epithelial cells infected by *F. nucleatum* with an MOI of 100 *via* high-throughput sequencing. Results showed the top 10 HUB genes were correlated with tumor progression. Moreover, some HUB genes were abnormally expressed in the clinical samples of OSCC. Kang et al. (2019) obtained the transcriptome map of gingival mesenchymal stem cells stimulated by *F. nucleatum via* gene chip significant map (maSigPro) analysis. After culture for 3, 7, 14 and 21 days, 790 (9 clusters) differentially expressed genes were found. These genes were significantly enriched in the cell adhesion junction and tumor-related pathways. The above-mentioned bioinformatics analysis showed that the infection of *F. nucleatum* in the oral cavity has a potential tumor-promoting effect. Harrandah et al. (2020) reported that compared uninfected mice, those infected with *F. nucleatum* and P. gingivalis developed significantly larger and numerous pathological changes in 4NQO-induced oral carcinoma in situ. Kamarajan et al. (2020) have found that the main periodontal pathogens (*P. gingivalis*, *T. denticola*, and *F. nucleatum*) can promote cell migration, invasion, tumorsphere formation, and OSCC tumorigenesis, without significantly affecting cell proliferation or apoptosis. Gallimidi et al. (Binder Gallimidi et al., 2015) reported that oral epithelial cells exposed to *P. gingivalis* and *F. nucleatum* can activate TLR signals, produce IL-6, activate STAT3, and induce important effector molecules (such as cyclin D1, MMP9, and heparanase) to drive the growth and invasion of OSCC. These results support the role of *F. nucleatum* in promoting OSCC. However, only few studies assessed the responsible mechanisms, which are still not well defined.

#### 6.2.1 Proliferation

In recent years, *F. nucleatum* have been found to promote the proliferation of OSCC. Uitto et al. (2005) reported that the upregulation of cyclin-dependent kinase (CDK) 7 and 9 mediated by *F. nucleatum* can enhance the proliferation of human immortalized keratinocytes. Geng et al. (2020) showed that *F. nucleatum* infection can cause DNA damage in tongue squamous cell carcinoma cell line Tca8113 *via* the Ku70/p53 pathway. This mechanism can then enhance proliferation ability and accelerate the cell cycle of Tca8113 cells. Although Ku70 participates in non-homologous end-joining of DNA repair by binding to the end of DNA double-strand breaks, the specific mechanism between *F. nucleatum* infection and Ku70 is unclear. Tumor suppressor protein p27 is a member of the CDK inhibitor family, which blocks cells from entering the S phase by binding to CDK and participates in cell cycle regulation. Geng et al. (2020) reported that the p27 levels in tongue squamous cell carcinoma cell lines infected by *F. nucleatum* decreased. Meanwhile, the percentage of cells in the G1 phase decreased, and that of cells in the S phase increased significantly. As previously mentioned, *F. nucleatum* can produce hydrogen sulfide. In this context, Ma et al. (2015) showed that hydrogen sulfide can accelerate the cell cycle process of OSCC cell lines. Zhang et al. (2016) revealed that hydrogen sulfide can promote the proliferation of oral cancer cells by activating the COX2/AKT/ERK1/2 axis. Hence, *F. nucleatum* may play a cancer-promoting role by producing hydrogen sulfide.

#### 6.2.2 Migration and Invasion

In addition to promoting the proliferation of OSCC, *F. nucleatum* may enhance its migration and invasion. Human epithelial cells infected with *F. nucleatum* can increase the production of MMP-9 and MMP-13 by activating mitogen-activated protein kinase p38 (Uitto et al., 2005). Meanwhile, MMP-9 and MMP-13 play an important role in tumor invasion and metastasis. Zhang et al. (Uitto et al., 2005) revealed that *F. nucleatum* can upregulate mesenchymal markers, including N-cadherin, vimentin and SNAI1, *via* the lncRNAMIR4435-2HG/miR-296-5p/Akt2/SNAI1 signal pathway in non-cancerous human immortalized oral epithelial cells and OSCC cell lines to promote the migration and epithelial-to-mesenchymal transition (EMT) of these two types of cells. Moreover, the promoting effect of *F. nucleatum* on EMT is not dependent on the whole living bacterial cells, and FadA may be closely correlated with this process. EMT refers to the biological process in which epithelial cells are transformed into cells with the interstitial phenotype *via* specific procedures. Epithelial-derived malignant tumors should have the ability to migrate and invade, which are important biological processes (Mittal, 2018). Kamarajan et al. (2020) reported that the migration of OSCC cells enhanced by the main periodontal pathogens (*P. gingivalis*, *T. denticola*, and *F. nucleatum*) is mediated by the activation of integrin αV and FAK, because the effect can be eliminated by the stable blocking of αV and FAK expression. Harrandah et al. (2020) showed that oral cancer cell lines infected with *F. nucleatum* upregulated the expression of MMP1, MMP9, and IL-8; MYC, JAK1, and STAT3, which are cell survival markers; and ZEB1 and TGF-β, which are EMT markers. Moreover, the culture supernatant of *F. nucleatum*, mainly LPS, was sufficient to induce IL-8 secretion, thereby indicating that living *F. nucleatum* may not require direct contact with cancer cells to change their behavior. Hence, the presence of *F. nucleatum* in the oral tumor microenvironment can potentially enhance the invasiveness, survival rate and EMT of cancer cells. The inflammasome contains a CARD (ASC), procaspase-1, and sensor protein, which is either a NOD-like receptor (NLR) or an absent in melanoma 2 (AIM2)-like receptor (Malik and Kanneganti, 2017). Furthermore, it can mediate the process of IL-1β, and pro-IL-18, which are the two most important inflammatory cytokines, to their active forms (Malik and Kanneganti, 2017). Aral et al. (2020) revealed that OSCC cell infection by *F. nucleatum* can enhance the expression of IL-1β by increasing AIM2 and by down-regulating POP1, which can control the activation of the NLRP3 inflammasome by targeting ASC. IL-1 β can participate in the early and late stages of oral carcinogenesis by promoting oral dysplastic cell proliferation, carcinogenic cytokine production, and OSCC invasiveness (Lee et al., 2015). Abdulkareem et al. (2018) reported that *F. nucleatum* may induce OSCC cells to undergo EMT by up-regulating TGF-β, TNF-α and EGF signals. However, at present, the specific molecular mechanisms of *F. nucleatum* promoting the migration and invasion of OSCC cells are not extremely clear, and most studies remained at the stage of *in vitro* experiments. Moreover, these findings have not been well confirmed in animal experiments.

#### 6.2.3 Change in the Local Tumor Microenvironment

**Figure 3:**
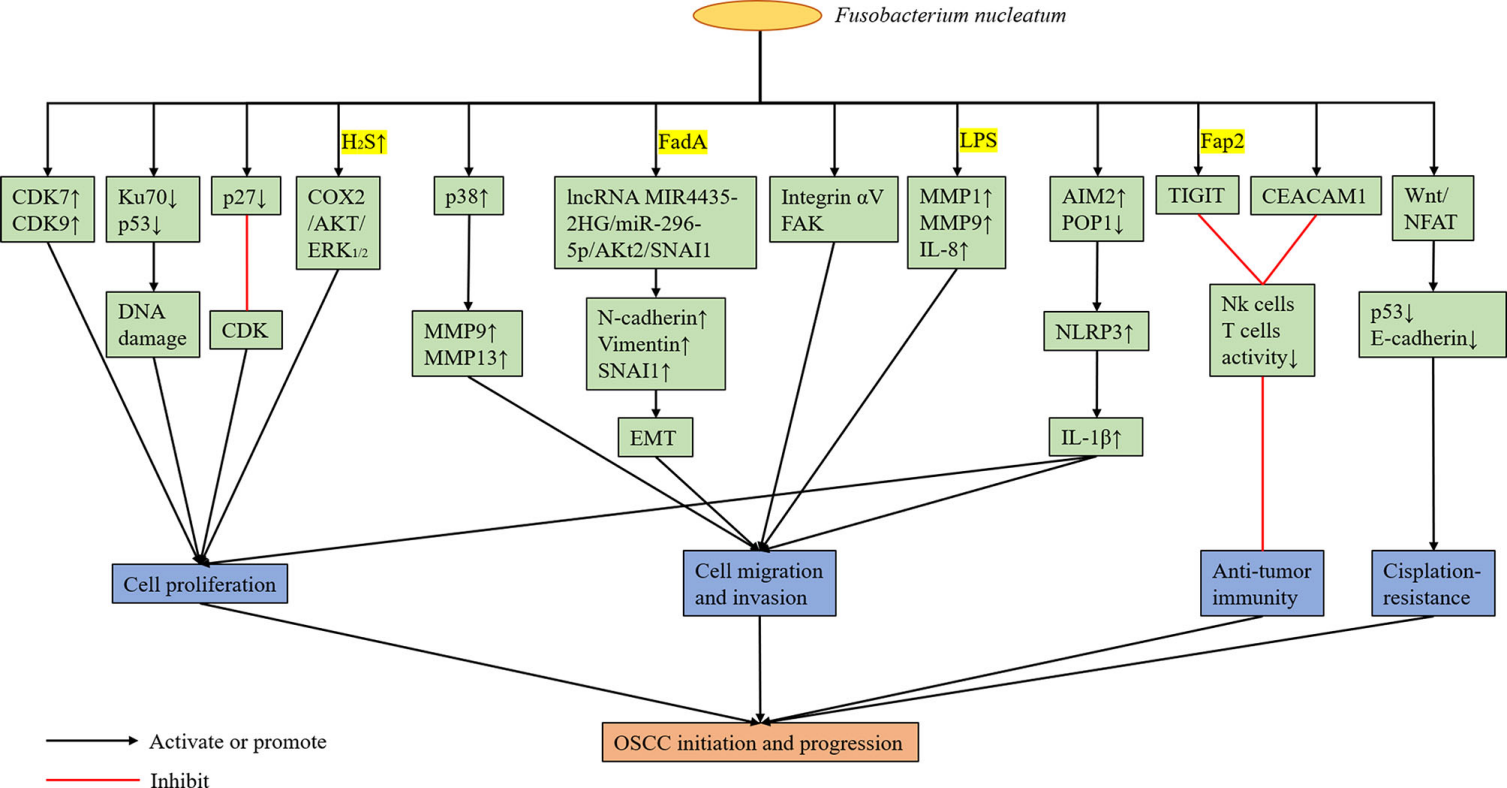
Possible Mechanisms of Cancer Promotion by *F. nucleatum*.

*F. nucleatum* can change the local immune microenvironment of the tumors it colonizes, and it plays a role in assisting tumor immune evasion. Gur et al. (2015) confirmed that the outer surface protein Fap2 of different *F. nucleatum* strains can bind and activate the human inhibitory receptor TIGIT expressed by T cells and natural killer cells (NK cells), thereby inhibiting anti-tumor immunity. Based on subsequent experiments, *F. nucleatum* was found to bind and activate the human inhibitory receptor CEACAM1. Hence, the activity of T cells and NK cells was inhibited (Gur et al., 2019). However, the specific protein of *F. nucleatum* binding to CEACAM1 is unknown. Therefore, *F. nucleatum* can promote the colonized tumor by regulating the immune microenvironment, which is beneficial to tumor development. In the future, drugs or CEACAM1 and TIGIT inhibitors targeting the surface proteins of *F. nucleatum* can be developed to eliminate bacterial-dependent tumor immune evasion and to help in the treatment of tumors colonized by *F. nucleatum*. In addition, *F. nucleatum* may have an adverse effect on the treatment of OSCC. Rui et al. (2021) found that *Fusobacterium* and *Mycoplasma* were more abundant in the nonresponsive group at the genus level *via* 16S rRNA gene sequencing and metagenomic analysis of oral rinse samples obtained from patients with OSCC who received docetaxel, cisplatin, and 5-fluorouracil (TPF) induction chemotherapy. Meanwhile, *Slackia* was more enriched in the responder group. Metagenomic shotgun sequencing analysis revealed that *F. nucleatum* was more enriched in the nonresponsive group (Rui et al., 2021). Therefore, *F. nucleatum* abundance may be correlated with poor response to chemotherapy in patients with OSCC. Da et al. (2021) showed that *F. nucleatum* may down-regulate p53 and E-cadherin *via* the Wnt/NFAT pathway, thereby promoting cisplatin resistance and OSCC cell migration. However, the molecular mechanism of the association between *F. nucleatum* and OSCC cells leading to cisplatin resistance has not been validated yet (**Figure 3**).

### 6.3 Anti-Cancer Treatment for *F. nucleatum*

Considering that bacterial infection may have a promoting effect in oral cancer, its treatment has been assessed in recent years. Kamarajan et al. (2015) found that Nisin can reduce the development of oral tumors, and its long-term use can prolong the life of tumor-bearing mice. Further, Nisin can eliminate cell migration, invasion, tumorsphere formation, and OSCC tumorigenesis promoted by *F. nucleatum in vivo* (Kamarajan et al., 2020). Therefore, Nisin has a good therapeutic potential and can be used as an anticancer agent and an inhibitor of pathogen-mediated carcinogenesis. With higher global antibiotic resistance rates, people are attempting to develop alternatives such as bacteriophages to achieve targeted therapy that attacks specific bacteria in biofilms and to prevent adverse consequences such as microbiota dysbiosis and antimicrobial resistance. Kabwe et al. (2019) identified a new type of bacteriophage FNUI against *F. nucleatum*, which can effectively kill cells in the biofilm of *F. nucleatum* and significantly reduce the number of *F. nucleatum* biofilms. Bacteriophages can be prepared in buccal tablets or pastes to kill potential bacteria when released *in vitro* (Brown et al., 2018). However, their use *in vivo* for the treatment of complex biofilms in periodontitis and as adjunctive therapy for cancer treatment must be further evaluated.

## 7 Association Between *F. nucleatum* and Systemic Diseases

**Figure 4:**
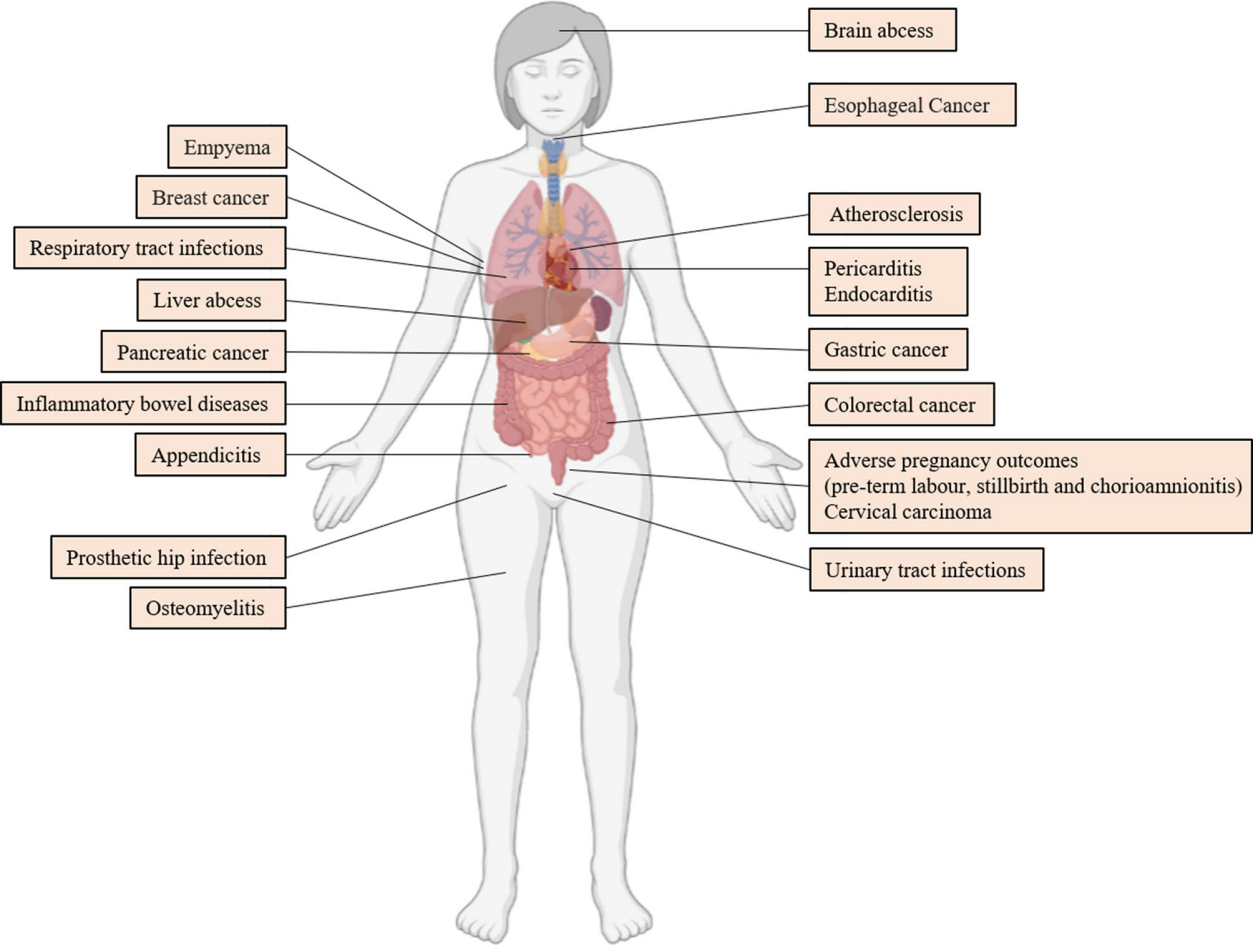
Extraoral Diseases Associated With *F. nucleatum*. References are (Truant et al., 1983; Ford et al., 2005; De Socio et al., 2009; Swidsinski et al., 2011; Lee et al., 2012; Mitsuhashi et al., 2015; Yamamura et al., 2016; Rodríguez Duque et al., 2018; Vander Haar et al., 2018; Hashemi Goradel et al., 2019; Liu et al., 2019; Abushamma et al., 2020; Barrera-López et al., 2020; Boehm et al., 2020; Cao et al., 2020; Huang et al., 2020; Kenig et al., 2020; Parhi et al., 2020; Swaminathan and Aguilar, 2020; Hoffmeister et al., 2021).

*F. nucleatum* is correlated with several diseases outside of the oral cavity. In this chapter, we reviewed the research progress of *F. nucleatum* in extra-oral diseases in recent years. **Figure 4** depicts the diseases in which *F. nucleatum* can be isolated from clinical specimens. However, whether *F. nucleatum* contributes to the development of these diseases must be validated. Therefore, this study assessed diseases including CRC, in which *F. nucleatum* has the most mechanistic supportive role, and adverse pregnancy outcomes (Brennan and Garrett, 2019).

### 7.1 CRC

*F. nucleatum* can be enriched in CRC tissues (Kostic et al., 2012), and patients with CRC had identical strains of *F. nucleatum* in the CRC and oral cavity (Komiya et al., 2019). Therefore, *F. nucleatum* in CRC may originate from the oral cavity.

*F. nucleatum* can affect the multiple stages of CRC development *via* different mechanisms (Brennan and Garrett, 2019). First, in the initial stage of tumorigenesis, *F. nucleatum* can activate the β-catenin and Wnt pathway *via* the binding of adhesion protein FadA to E-cadherin on the surface of CRC cells (Rubinstein et al., 2013). This phenomenon leads to the activation of the NF-κB pathway *via* the combination of virulence factor LPS and TLR-4 on the surface of CRC cells, which results in a higher carcinogenic miR-21 expression (Yang et al., 2017). The above-mentioned mechanisms can promote the proliferation of cancer cells.

Second, if a tumor develops, *F. nucleatum* can bind to acetylgalactosamine (Gal-GalNAc) overexpressed on the surface of CRC cells *via* fibroblast activation protein 2 (Fap2) lectins on its surface, thereby resulting in the local enrichment of *F. nucleatum* in cancer tissues (Yang et al., 2017). In addition, *F. nucleatum* can bind to the colon epithelium *via* FadA and RadD and invade the mucosa (Wu et al., 2019).

Third, in the process of tumor development, *F. nucleatum* can induce a pro-inflammatory microenvironment and suppress host immunity conducive to CRC progression (Wu et al., 2019). The invasion of *F. nucleatum* increases the infiltration of inflammatory cells and the release of cytokines, such as NF-κB, IL-6, IL-8, IL-10, and IL-18, which promote cell proliferation (Wu et al., 2019). Moreover, it can interact with the immune cells, leading to a lower T cell density, greater M2 macrophage polarization, NK cell activity inhibition, and higher number of dendritic cells and tumor-associated neutrophils that diminish anti-tumor immunity (Wu et al., 2019). Moreover, it can selectively recruit tumor-infiltrating myeloid cells, which can promote tumor progression (Kostic et al., 2013). In addition to forming a local cancer-promoting immune microenvironment, *F. nucleatum* can promote CRC metastasis. *F. nucleatum* infection can upregulate caspase recruitment domain 3 (CARD3) expression by activating autophagy signaling (Chen et al., 2020) and by upregulating *KRT7-AS/KRT7* by activating the NF-κB pathway (Chen et al., 2020), which promotes CRC metastasis. Moreover, *F. nucleatum* infection can increase the secretion of miR-1246/92b-3p/27a-3p and CXCL16/RhoA/IL-8-enriched exosomes from CRC cells, which can enhance the cell migration ability of non-infected CRC cells *in vitro* and promote CRC metastasis *in vivo* (Guo et al., 2020).

Fourth, in the tumor treatment stage, *F. nucleatum* can increase the risk of recurrence and chemotherapy resistance by inhibiting the specific miRNAs involved in autophagy (Yu et al., 2017). Moreover, it can migrate to the CRC locus, impair the therapeutic efficacy of radiotherapy, and affect patient prognosis. The use of metronidazole to kill F. nucleatum can reduce CRC radiotherapy resistance induced by oral microbiota (Dong et al., 2021). To enhance the chemotherapy effect of CRC, Zheng et al. (2019) linked the *F. nucleatum*-specific phage with dextran nanoparticles loaded with CRC chemotherapeutic drugs to form phage-guided nanoparticles, which can effectively inhibit the growth of *F. nucleatum*, significantly prolong the survival time of CRC mice and reduce the number of adenomas in Apc mice prone to intestinal adenomas. These results indicate that phage-guided nanotechnology provides a new method for the future treatment of CRC. A recent study showed that in patients with CRC, the abundance of *F. nucleatum* is positively correlated with a high glucose metabolism of CRC cells. *F. nucleatum* regulates the histone modification of ENO1 (a key component of glycolysis pathway) gene by upregulating lncRNAENO1-IT1, thereby promoting the glycolysis of CRC cells (which provide energy for tumor cells) and cancer (Hong et al., 2021). Therefore, targeting the ENO1 pathway can be a potential therapeutic strategy in patients with CRC with a high abundance of *F. nucleatum*.

Recent studies have shown that *F. nucleatum* can promote the development of CRC *via* different mechanisms. However, at present, whether *F. nucleatum* is a cause or result of CRC has not been elucidated. Regardless, *F. nucleatum* should be considered a risk factor for CRC, and targeting *F. nucleatum* in the treatment of CRC may help improve the prognosis of patients with CRC.

### 7.2 Adverse Pregnancy Outcomes

In recent years, using 16S rRNA-based culture-independent methods, researchers have found *F. nucleatum* in placental and fetal tissues (Cahill et al., 2005; Han et al., 2009; Gonzales-Marin et al., 2013; Wang et al., 2013). Previous studies have shown that *F. nucleatum* in the placenta may originate from subgingival plaque in the oral cavity. *Via* the identification of clinical samples, the strains of *F. nucleatum* found in the amniotic fluid and placenta matched the strains in the maternal or paternal’s subgingival regions, rather than in the lower genital tract (Gauthier et al., 2011). Animal studies have revealed that the injection of saliva or subgingival plaque samples into mice can cause infection caused by oral symbiotic species, including *F. nucleatum*, in the murine placenta. Therefore, oral bacteria can translocate to the fetal-placental unit (Fardini et al., 2010).

Animal models have shown the pathogenic role of *F. nucleatum* in adverse pregnancy outcomes. Han et al. (2004) found that the injection of *F. nucleatum* into the tail vein of pregnant mice caused preterm and term fetal loss within 72 h. Further, *F. nucleatum* was restricted inside the uterus, without spreading systemically. Aother study found that *F. nucleatum* induced fetal death in mice *via* the stimulation of TLR4-mediated placental inflammatory response (Liu et al., 2007). The ability of *F. nucleatum* to colonize the mouse placenta is closely correlated with the adhesins on its surface (Han et al., 2005; Kaplan et al., 2009; Kaplan et al., 2014; Coppenhagen-Glazer et al., 2015). Among them, FadA plays a critical role in the murine model of infection (Han, 2015). When FadA binds to vascular endothelial cadherin (VE-cadherin), it causes VE-cadherin to migrate away from the cell-cell junction, thereby increasing endothelial permeability and allowing microorganisms to penetrate the endothelium (Fardini et al., 2011). These mechanisms can explain how *F. nucleatum* overcomes obstacles including the placental barrier. Therefore, this finding supports the importance of oral health and dental care among pregnant women.

## 8 Summary and Conclusions

*F. nucleatum*, which is one of the main pathogens associated with periodontal diseases, not only causes periodontal disease and halitosis, but also plays an important role in dental pulp infection. In addition, a large body of epidemiological data shows that it is also essential in promoting OSCC and is considered a carcinogenic bacterium. Although periodontitis and cancer are different diseases, their associated wounds do not heal (Cugini et al., 2013). Periodontitis is an independent risk factor for OSCC (Tezal et al., 2009). Moreover, they have potential similarities in terms of the pathogenic mechanisms of *F. nucleatum* in both diseases. *F. nucleatum* can play a role in these two diseases by causing chronic inflammation, promoting EMT of epithelial cells, and altering the local immune microenvironment.

In addition, the specific mechanisms of oral carcinogenic bacteria including *F. nucleatum* in OSCC are still not completely understood. It may be extremely early to consider eliminating carcinogenic bacteria to prevent oral cancer. Drugs that can effectively target pathogenic bacteria are still being investigated. In the future, with further development of bacteria detection technology and large data analysis, a spectrum of carcinogenic bacteria in terms of different regions, races, ages, and stages of tumor development can be established based on large population data. Determining the diagnostic biomarkers for OSCC at an early stage is extremely significant for the prevention and early diagnosis and treatment of oral cancer and the identification of prognosis. In the future, tumoral microorganisms can be used as a basis for prognosis and treatment decision-makings, and microbial profiling may soon become a routine test for OSCC.

*F. nucleatum* can be detected in different extra-oral diseases. However, except in CRC and adverse pregnancy outcomes, its role in other diseases and specific pathogenic mechanisms remains unknown. With further research development, we can have a better understanding of *F. nucleatum*, which lays the foundation for the development of prevention and treatment strategies for related diseases.

## Author Contributions

YC, ZH, BJ, and JZ wrote the manuscript. ZT, YH, MH, and HL made the figures and edited the manuscript. DZ, GS, BJ, and JZ administrated and supervised the whole research project. All authors have read and agreed to the published version of the manuscript.

## Funding

This research was supported by the National Natural Science Foundation of China (grant numbers 81670950).

## Conflict of Interest

The authors declare that the research was conducted in the absence of any commercial or financial relationships that could be construed as a potential conflict of interest.

## Publisher’s Note

All claims expressed in this article are solely those of the authors and do not necessarily represent those of their affiliated organizations, or those of the publisher, the editors and the reviewers. Any product that may be evaluated in this article, or claim that may be made by its manufacturer, is not guaranteed or endorsed by the publisher.

## References

[B1] Abdulkareem A. A. Shelton R. M. Landini G. Cooper P. R. Milward M. R. (2018a). Potential Role of Periodontal Pathogens in Compromising Epithelial Barrier Function by Inducing Epithelial-Mesenchymal Transition. J. Periodontal. Res. 53 (4), 565–574. doi: 10.1111/jre.12546 29704258

[B2] Abdulkareem A. A. Shelton R. M. Landini G. Cooper P. R. Milward M. R. (2018b). Periodontal Pathogens Promote Epithelial-Mesenchymal Transition in Oral Squamous Carcinoma Cells *In Vitro* . Cell Adh. Migr. 12 (2), 127–137. doi: 10.1080/19336918.2017.1322253 28873015 PMC5927641

[B3] Abushamma F. Perry-Thomas R. Hammond C. Horsch A. D. Whittlestone T. (2020). Xanthogranulomatous Pyelonephritis Caused by Fusobacterium Nucleatum. Case Report and Review of Literature. Urol. Case Rep. 33, 101293. doi: 10.1016/j.eucr.2020.101293 33101996 PMC7573844

[B4] Adedapo A. H. Kolude B. Dada-Adegbola H. O. Lawoyin J. O. Adeola H. A. (2020). Targeted Polymerase Chain Reaction-Based Expression of Putative Halitogenic Bacteria and Volatile Sulphur Compound Analysis Among Halitosis Patients at a Tertiary Hospital in Nigeria. Odontology 108 (3), 450–461. doi: 10.1007/s10266-019-00467-x 31641894

[B5] Al-Hebshi N. N. Nasher A. T. Maryoud M. Y. Homeida H. E. Chen T. Idris A. M.. (2017). Inflammatory Bacteriome Featuring Fusobacterium Nucleatum and Pseudomonas Aeruginosa Identified in Association With Oral Squamous Cell Carcinoma. Sci. Rep. 7 (1), 1834. doi: 10.1038/s41598-017-02079-3 28500338 PMC5431832

[B6] Altshuler G. Hyde S. (1988). Clinicopathologic Considerations of Fusobacteria Chorioamnionitis. Acta Obstet. Gynecol. Scand. 67 (6), 513–517. doi: 10.3109/00016348809029862 3071072

[B7] Amou T. Hinode D. Yoshioka M. Grenier D. (2014). Relationship Between Halitosis and Periodontal Disease - Associated Oral Bacteria in Tongue Coatings. Int. J. Dent. Hyg 12 (2), 145–151. doi: 10.1111/idh.12046 23890391

[B8] Aral K. Milward M. R. Gupta D. Cooper P. R. (2020). Effects of Porphyromonas Gingivalis and Fusobacterium Nucleatum on Inflammasomes and Their Regulators in H400 Cells. Mol. Oral. Microbiol. 35 (4), 158–167. doi: 10.1111/omi.12302 32516848

[B9] Arenas Rodrigues V. A. de Avila E. D. Nakano V. Avila-Campos M. J. (2018). Qualitative, Quantitative and Genotypic Evaluation of Aggregatibacter Actinomycetemcomitans and Fusobacterium Nucleatum Isolated From Individuals With Different Periodontal Clinical Conditions. Anaerobe 52, 50–58. doi: 10.1016/j.anaerobe.2018.05.015 29857043

[B10] Bachrach G. Rosen G. Bellalou M. Naor R. Sela M. N. (2004). Identification of a Fusobacterium Nucleatum 65 kDa Serine Protease. Oral. Microbiol. Immunol. 19 (3), 155–159. doi: 10.1111/j.0902-0055.2004.00132.x 15107066

[B11] Barbosa-Ribeiro M. Arruda-Vasconcelos R. Louzada L. M. Dos Santos D. G. Andreote F. D. Gomes B. (2021). Microbiological Analysis of Endodontically Treated Teeth With Apical Periodontitis Before and After Endodontic Retreatment. Clin. Oral. Investig. 25 (4), 2017–2027. doi: 10.1007/s00784-020-03510-2 32860137

[B12] Barrera-López L. Macía-Rodríguez C. Ferreiro-Fernández L. Díaz-Peromingo J. A. (2020). Fusobacterium Nucleatum Empyema: An Atypical Presentation. Eur. J. Case Rep. Intern. Med. 7 (7), 001631. doi: 10.12890/2020_001631 32665930 PMC7350953

[B13] Basic A. Blomqvist M. Dahlén G. Svensäter G. (2017). The Proteins of Fusobacterium Spp. Involved in Hydrogen Sulfide Production From L-Cysteine. BMC Microbiol. 17 (1), 61. doi: 10.1186/s12866-017-0967-9 28288582 PMC5348791

[B14] Ben Lagha A. Vaillancourt K. Maquera Huacho P. Grenier D. (2020). Effects of Labrador Tea, Peppermint, and Winter Savory Essential Oils on Fusobacterium Nucleatum. Antibiotics (Basel) 9 (11), 794. doi: 10.3390/antibiotics9110794 PMC7697736 33182686

[B15] Bernardi S. Continenza M. A. Al-Ahmad A. Karygianni L. Follo M. Filippi A.. (2019). Streptococcus Spp. And Fusobacterium Nucleatum in Tongue Dorsum Biofilm From Halitosis Patients: A Fluorescence *in Situ* Hybridization (FISH) and Confocal Laser Scanning Microscopy (CLSM) Study. New Microbiol. 42 (2), 108–113. 31034083

[B16] Binder Gallimidi A. Fischman S. Revach B. Bulvik R. Maliutina A. Rubinstein A. M.. (2015). Periodontal Pathogens Porphyromonas Gingivalis and Fusobacterium Nucleatum Promote Tumor Progression in an Oral-Specific Chemical Carcinogenesis Model. Oncotarget 6 (26), 22613–22623. doi: 10.18632/oncotarget.4209 26158901 PMC4673186

[B17] Boehm E. T. Thon C. Kupcinskas J. Steponaitiene R. Skieceviciene J. Canbay A.. (2020). Fusobacterium Nucleatum Is Associated With Worse Prognosis in Lauren’s Diffuse Type Gastric Cancer Patients. Sci. Rep. 10 (1), 16240. doi: 10.1038/s41598-020-73448-8 33004953 PMC7530997

[B18] Bolstad A. I. Jensen H. B. Bakken V. (1996). Taxonomy, Biology, and Periodontal Aspects of Fusobacterium Nucleatum. Clin. Microbiol. Rev. 9 (1), 55–71. doi: 10.1128/CMR.9.1.55 8665477 PMC172882

[B19] Bouillaguet S. Manoil D. Girard M. Louis J. Gaïa N. Leo S.. (2018). Root Microbiota in Primary and Secondary Apical Periodontitis. Front. Microbiol. 9, 2374. doi: 10.3389/fmicb.2018.02374 30356779 PMC6189451

[B20] Boyce B. F. Li P. Yao Z. Zhang Q. Badell I. R. Schwarz E. M.. (2005). TNF-Alpha and Pathologic Bone Resorption. Keio J. Med. 54 (3), 127–131. doi: 10.2302/kjm.54.127 16237274

[B21] Brennan C. A. Garrett W. S. (2019). Fusobacterium Nucleatum - Symbiont, Opportunist and Oncobacterium. Nat. Rev. Microbiol. 17 (3), 156–166. doi: 10.1038/s41579-018-0129-6 30546113 PMC6589823

[B22] Brown T. L. Petrovski S. Chan H. T. Angove M. J. Tucci J. (2018). Semi-Solid and Solid Dosage Forms for the Delivery of Phage Therapy to Epithelia. Pharm. (Basel) 11 (1), 26. doi: 10.3390/ph11010026 PMC5874722 29495355

[B23] Cahill R. J. Tan S. Dougan G. O’Gaora P. Pickard D. Kennea N.. (2005). Universal DNA Primers Amplify Bacterial DNA From Human Fetal Membranes and Link Fusobacterium Nucleatum With Prolonged Preterm Membrane Rupture. Mol. Hum. Reprod. 11 (10), 761–766. doi: 10.1093/molehr/gah234 16254004

[B24] Cao P. Chen Y. Guo X. Chen Y. Su W. Zhan N.. (2020). Fusobacterium Nucleatum Activates Endoplasmic Reticulum Stress to Promote Crohn’s Disease Development *via* the Upregulation of CARD3 Expression. Front. Pharmacol. 11, 106. doi: 10.3389/fphar.2020.00106 32153411 PMC7047714

[B25] Chang M. C. Chen Y. J. Lian Y. C. Chang B. E. Huang C. C. Huang W. L.. (2018). Butyrate Stimulates Histone H3 Acetylation, 8-Isoprostane Production, RANKL Expression, and Regulated Osteoprotegerin Expression/Secretion in MG-63 Osteoblastic Cells. Int. J. Mol. Sci. 19 (12). doi: 10.3390/ijms19124071 PMC6321057 30562925

[B26] Chang C. Geng F. Shi X. Li Y. Zhang X. Zhao X.. (2019). The Prevalence Rate of Periodontal Pathogens and its Association With Oral Squamous Cell Carcinoma. Appl. Microbiol. Biotechnol. 103 (3), 1393–1404. doi: 10.1007/s00253-018-9475-6 30470868

[B27] Chaushu S. Wilensky A. Gur C. Shapira L. Elboim M. Halftek G.. (2012). Direct Recognition of Fusobacterium Nucleatum by the NK Cell Natural Cytotoxicity Receptor NKp46 Aggravates Periodontal Disease. PloS Pathog. 8 (3), e1002601. doi: 10.1371/journal.ppat.1002601 22457623 PMC3310798

[B28] Chávez de Paz L. E. Bergenholtz G. Dahlén G. Svensäter G. (2007). Response to Alkaline Stress by Root Canal Bacteria in Biofilms. Int. Endod. J. 40 (5), 344–355. doi: 10.1111/j.1365-2591.2006.01226.x 17326786

[B29] Chen Y. Chen Y. Zhang J. Cao P. Su W. Deng Y.. (2020). Fusobacterium Nucleatum Promotes Metastasis in Colorectal Cancer by Activating Autophagy Signaling *via* the Upregulation of CARD3 Expression. Theranostics 10 (1), 323–339. doi: 10.7150/thno.38870 31903123 PMC6929621

[B30] Chen S. Su T. Zhang Y. Lee A. He J. Ge Q.. (2020). Fusobacterium Nucleatum Promotes Colorectal Cancer Metastasis by Modulating KRT7-As/Krt7. Gut Microbes 11 (3), 511–525. doi: 10.1080/19490976.2019.1695494 31910722 PMC7524269

[B31] Coppenhagen-Glazer S. Sol A. Abed J. Naor R. Zhang X. Han Y. W.. (2015). Fap2 of Fusobacterium Nucleatum is a Galactose-Inhibitable Adhesin Involved in Coaggregation, Cell Adhesion, and Preterm Birth. Infect. Immun. 83 (3), 1104–1113. doi: 10.1128/IAI.02838-14 25561710 PMC4333458

[B32] Cugini C. Klepac-Ceraj V. Rackaityte E. Riggs J. E. Davey M. E. (2013). Porphyromonas Gingivalis: Keeping the Pathos Out of the Biont. J. Oral. Microbiol. 5. doi: 10.3402/jom.v5i0.19804 PMC3617648 23565326

[B33] Da J. Wang X. Li L. Xu Y. (2021). Fusobacterium Nucleatum Promotes Cisplatin-Resistance and Migration of Oral Squamous Carcinoma Cells by Up-Regulating Wnt5a-Mediated NFATc3 Expression. Tohoku J. Exp. Med. 253 (4), 249–259. doi: 10.1620/tjem.253.249 33840648

[B34] de Andrade K. Q. Almeida-da-Silva C. L. C. Coutinho-Silva R. (2019). Immunological Pathways Triggered by Porphyromonas Gingivalis and Fusobacterium Nucleatum: Therapeutic Possibilities? Mediators Inflamm. 2019, 7241312. doi: 10.1155/2019/7241312 31341421 PMC6612971

[B35] De Lima P. O. Nani B. D. Rolim G. S. Groppo F. C. Franz-Montan M. Alves De Moraes A. B.. (2020). Effects of Academic Stress on the Levels of Oral Volatile Sulfur Compounds, Halitosis-Related Bacteria and Stress Biomarkers of Healthy Female Undergraduate Students. J. Breath Res. 14 (3), 036005. doi: 10.1088/1752-7163/ab944d 32428892

[B36] De Rossi A. Rocha L. B. Rossi M. A. (2008). Interferon-Gamma, Interleukin-10, Intercellular Adhesion Molecule-1, and Chemokine Receptor 5, But Not Interleukin-4, Attenuate the Development of Periapical Lesions. J. Endod. 34 (1), 31–38. doi: 10.1016/j.joen.2007.09.021 18155488

[B37] De Socio G. V. Mencacci A. Bini P. Pasticci M. B. (2009). Fusobacterium Nucleatum Endocarditis Mimicking Polymyalgia Rheumatica. South Med. J. 102 (10), 1082–1084. doi: 10.1097/SMJ.0b013e3181b4e5b8 19738533

[B38] Didilescu A. C. Rusu D. Anghel A. Nica L. Iliescu A. Greabu M.. (2012). Investigation of Six Selected Bacterial Species in Endo-Periodontal Lesions. Int. Endod. J. 45 (3), 282–293. doi: 10.1111/j.1365-2591.2011.01974.x 22077868

[B39] Dong J. Li Y. Xiao H. Zhang S. Wang B. Wang H.. (2021). Oral Microbiota Affects the Efficacy and Prognosis of Radiotherapy for Colorectal Cancer in Mouse Models. Cell Rep. 37 (4), 109886. doi: 10.1016/j.celrep.2021.109886 34706245

[B40] D’Souza S. Addepalli V. (2018). Preventive Measures in Oral Cancer: An Overview. BioMed. Pharmacother. 107, 72–80. doi: 10.1016/j.biopha.2018.07.114 30081204

[B41] Fardini Y. Chung P. Dumm R. Joshi N. Han Y. W. (2010). Transmission of Diverse Oral Bacteria to Murine Placenta: Evidence for the Oral Microbiome as a Potential Source of Intrauterine Infection. Infect. Immun. 78 (4), 1789–1796. doi: 10.1128/IAI.01395-09 20123706 PMC2849412

[B42] Fardini Y. Wang X. Témoin S. Nithianantham S. Lee D. Shoham M.. (2011). Fusobacterium Nucleatum Adhesin FadA Binds Vascular Endothelial Cadherin and Alters Endothelial Integrity. Mol. Microbiol. 82 (6), 1468–1480. doi: 10.1111/j.1365-2958.2011.07905.x 22040113 PMC3237733

[B43] Ford P. J. Gemmell E. Hamlet S. M. Hasan A. Walker P. J. West M. J.. (2005). Cross-Reactivity of GroEL Antibodies With Human Heat Shock Protein 60 and Quantification of Pathogens in Atherosclerosis. Oral. Microbiol. Immunol. 20 (5), 296–302. doi: 10.1111/j.1399-302X.2005.00230.x 16101965

[B44] Fujiwara N. Murakami K. Nakao M. Toguchi M. Yumoto H. Amoh T.. (2017). Novel Reuterin-Related Compounds Suppress Odour by Periodontopathic Bacteria. Oral. Dis. 23 (4), 492–497. doi: 10.1111/odi.12638 28083982

[B45] Fukada S. Y. Silva T. A. Garlet G. P. Rosa A. L. da Silva J. S. Cunha F. Q. (2009). Factors Involved in the T Helper Type 1 and Type 2 Cell Commitment and Osteoclast Regulation in Inflammatory Apical Diseases. Oral. Microbiol. Immunol. 24 (1), 25–31. doi: 10.1111/j.1399-302X.2008.00469.x 19121066

[B46] Gao Y. Grassi F. Ryan M. R. Terauchi M. Page K. Yang X.. (2007). IFN-Gamma Stimulates Osteoclast Formation and Bone Loss *In Vivo via* Antigen-Driven T Cell Activation. J. Clin. Invest. 117 (1), 122–132. doi: 10.1172/JCI30074 17173138 PMC1697800

[B47] Gauthier S. Tétu A. Himaya E. Morand M. Chandad F. Rallu F.. (2011). The Origin of Fusobacterium Nucleatum Involved in Intra-Amniotic Infection and Preterm Birth. J. Matern. Fetal Neonatal Med. 24 (11), 1329–1332. doi: 10.3109/14767058.2010.550977 21314291

[B48] Geng F. Zhang Y. Lu Z. Zhang S. Pan Y. (2020). Fusobacterium Nucleatum Caused DNA Damage and Promoted Cell Proliferation by the Ku70/p53 Pathway in Oral Cancer Cells. DNA Cell Biol. 39 (1), 144–151. doi: 10.1089/dna.2019.5064 31765243 PMC6978777

[B49] Gomes B. P. Endo M. S. Martinho F. C. (2012). Comparison of Endotoxin Levels Found in Primary and Secondary Endodontic Infections. J. Endod. 38 (8), 1082–1086. doi: 10.1016/j.joen.2012.04.021 22794210

[B50] Gonzales-Marin C. Spratt D. A. Allaker R. P. (2013). Maternal Oral Origin of Fusobacterium Nucleatum in Adverse Pregnancy Outcomes as Determined Using the 16S-23S rRNA Gene Intergenic Transcribed Spacer Region. J. Med. Microbiol. 62 (Pt 1), 133–144. doi: 10.1099/jmm.0.049452-0 23002071

[B51] Gregory S. W. Boyce T. G. Larson A. N. Patel R. Jackson M. A. (2015). Fusobacterium Nucleatum Osteomyelitis in 3 Previously Healthy Children: A Case Series and Review of the Literature. J. Pediatr. Infect. Dis. Soc. 4 (4), e155–e159. doi: 10.1093/jpids/piv052 PMC4681383 26407282

[B52] Guo S. Chen J. Chen F. Zeng Q. Liu W. L. Zhang G. (2020). Exosomes Derived From Fusobacterium Nucleatum-Infected Colorectal Cancer Cells Facilitate Tumour Metastasis by Selectively Carrying miR-1246/92b-3p/27a-3p and CXCL16. Gut 2020. doi: 10.1136/gutjnl-2020-321187 33172926

[B53] Guo L. Shokeen B. He X. Shi W. Lux R. (2017). Streptococcus Mutans SpaP Binds to RadD of Fusobacterium Nucleatum Ssp. Polymorphum. Mol. Oral. Microbiol. 32 (5), 355–364. doi: 10.1111/omi.12177 27976528 PMC5472499

[B54] Gur C. Ibrahim Y. Isaacson B. Yamin R. Abed J. Gamliel M.. (2015). Binding of the Fap2 Protein of Fusobacterium Nucleatum to Human Inhibitory Receptor TIGIT Protects Tumors From Immune Cell Attack. Immunity 42 (2), 344–355. doi: 10.1016/j.immuni.2015.01.010 25680274 PMC4361732

[B55] Gur C. Maalouf N. Shhadeh A. Berhani O. Singer B. B. Bachrach G.. (2019). Fusobacterium Nucleatum Supresses Anti-Tumor Immunity by Activating CEACAM1. Oncoimmunology 8 (6), e1581531. doi: 10.1080/2162402X.2019.1581531 31069151 PMC6492956

[B56] Hampelska K. Jaworska M. M. Babalska Z. Karpiński T. M. (2020). The Role of Oral Microbiota in Intra-Oral Halitosis. J. Clin. Med. 9 (8), 2484. doi: 10.3390/jcm9082484 PMC7465478 32748883

[B57] Han Y. W. (2015). Fusobacterium Nucleatum: A Commensal-Turned Pathogen. Curr. Opin. Microbiol. 23, 141–147. doi: 10.1016/j.mib.2014.11.013 25576662 PMC4323942

[B58] Han Y. W. Ikegami A. Rajanna C. Kawsar H. I. Zhou Y. Li M.. (2005). Identification and Characterization of a Novel Adhesin Unique to Oral Fusobacteria. J. Bacteriol. 187 (15), 5330–5340. doi: 10.1128/JB.187.15.5330-5340.2005 16030227 PMC1196005

[B59] Han Y. W. Redline R. W. Li M. Yin L. Hill G. B. McCormick T. S. (2004). Fusobacterium Nucleatum Induces Premature and Term Stillbirths in Pregnant Mice: Implication of Oral Bacteria in Preterm Birth. Infect. Immun. 72 (4), 2272–2279. doi: 10.1128/IAI.72.4.2272-2279.2004 15039352 PMC375172

[B60] Han Y. W. Shen T. Chung P. Buhimschi I. A. Buhimschi C. S. (2009). Uncultivated Bacteria as Etiologic Agents of Intra-Amniotic Inflammation Leading to Preterm Birth. J. Clin. Microbiol. 47 (1), 38–47. doi: 10.1128/JCM.01206-08 18971361 PMC2620857

[B61] Han X. Y. Weinberg J. S. Prabhu S. S. Hassenbusch S. J. Fuller G. N. Tarrand J. J.. (2003). Fusobacterial Brain Abscess: A Review of Five Cases and an Analysis of Possible Pathogenesis. J. Neurosurg. 99 (4), 693–700. doi: 10.3171/jns.2003.99.4.0693 14567605

[B62] Harrandah A. M. Chukkapalli S. S. Bhattacharyya I. Progulske-Fox A. Chan E. K. L. (2020). Fusobacteria Modulate Oral Carcinogenesis and Promote Cancer Progression. J. Oral. Microbiol. 13 (1), 1849493. doi: 10.1080/20002297.2020.1849493 33391626 PMC7717872

[B63] Hashemi Goradel N. Heidarzadeh S. Jahangiri S. Farhood B. Mortezaee K. Khanlarkhani N.. (2019). Fusobacterium Nucleatum and Colorectal Cancer: A Mechanistic Overview. J. Cell Physiol. 234 (3), 2337–2344. doi: 10.1002/jcp.27250 30191984

[B64] He J. Huang W. Pan Z. Cui H. Qi G. Zhou X.. (2012). Quantitative Analysis of Microbiota in Saliva, Supragingival, and Subgingival Plaque of Chinese Adults With Chronic Periodontitis. Clin. Oral. Investig. 16 (6), 1579–1588. doi: 10.1007/s00784-011-0654-4 22169888

[B65] Higuchi T. Suzuki N. Nakaya S. Omagari S. Yoneda M. Hanioka T.. (2019). Effects of Lactobacillus Salivarius WB21 Combined With Green Tea Catechins on Dental Caries, Periodontitis, and Oral Malodor. Arch. Oral. Biol. 98, 243–247. doi: 10.1016/j.archoralbio.2018.11.027 30530235

[B66] Hoffmeister B. C. Ducasse C. K. González L. M. Quilodrán S. C. Joyas M. A. (2021). Pulmonary and Thoracic Infection by Fusobacterium Nucleatum. Andes Pediatr. 92 (1), 93–98. doi: 10.32641/andespediatr.v92i1.1744 34106188

[B67] Hong J. Guo F. Lu S. Y. Shen C. Ma D. Zhang X.. (2021). F. Nucleatum Targets lncRNA ENO1-IT1 to Promote Glycolysis and Oncogenesis in Colorectal Cancer. Gut 70 (11), 2123–2137. doi: 10.1136/gutjnl-2020-322780 33318144

[B68] Hosgood H. D. Cai Q. Hua X. Long J. Shi J. Wan Y.. (2021). Variation in Oral Microbiome is Associated With Future Risk of Lung Cancer Among Never-Smokers. Thorax 76 (3), 256–263. doi: 10.1136/thoraxjnl-2020-215542 33318237 PMC8513501

[B69] Hsieh Y. Y. Tung S. Y. Pan H. Y. Yen C. W. Xu H. W. Lin Y. J.. (2018). Increased Abundance of Clostridium and Fusobacterium in Gastric Microbiota of Patients With Gastric Cancer in Taiwan. Sci. Rep. 8 (1), 158. doi: 10.1038/s41598-017-18596-0 29317709 PMC5760541

[B70] Huang S. T. Chen J. Lian L. Y. Cai H. H. Zeng H. S. Zheng M.. (2020). Intratumoral Levels and Prognostic Significance of Fusobacterium Nucleatum in Cervical Carcinoma. Aging (Albany NY) 12 (22), 23337–23350. doi: 10.18632/aging.104188 33197886 PMC7746363

[B71] Hung S. C. Huang P. R. Almeida-da-Silva C. L. C. Atanasova K. R. Yilmaz O. Ojcius D. M. (2018). NLRX1 Modulates Differentially NLRP3 Inflammasome Activation and NF-κb Signaling During Fusobacterium Nucleatum Infection. Microbes Infect. 20 (9-10), 615–625. doi: 10.1016/j.micinf.2017.09.014 29024797 PMC5891395

[B72] Ito S. Shimura S. Tanaka T. Yaegaki K. (2010). Myrsinoic Acid B Inhibits the Production of Hydrogen Sulfide by Periodontal Pathogens *In Vitro* . J. Breath Res. 4 (2), 026005. doi: 10.1088/1752-7155/4/2/026005 21383473

[B73] Johnson L. Almeida-da-Silva C. L. C. Takiya C. M. Figliuolo V. Rocha G. M. Weissmüller G.. (2018). Oral Infection of Mice With Fusobacterium Nucleatum Results in Macrophage Recruitment to the Dental Pulp and Bone Resorption. BioMed. J. 41 (3), 184–193. doi: 10.1016/j.bj.2018.05.001 30080658 PMC6138822

[B74] Johnson E. M. Flannagan S. E. Sedgley C. M. (2006). Coaggregation Interactions Between Oral and Endodontic Enterococcus Faecalis and Bacterial Species Isolated From Persistent Apical Periodontitis. J. Endod. 32 (10), 946–950. doi: 10.1016/j.joen.2006.03.023 16982270

[B75] Kabwe M. Brown T. L. Dashper S. Speirs L. Ku H. Petrovski S.. (2019). Genomic, Morphological and Functional Characterisation of Novel Bacteriophage FNU1 Capable of Disrupting Fusobacterium Nucleatum Biofilms. Sci. Rep. 9 (1), 9107. doi: 10.1038/s41598-019-45549-6 31235721 PMC6591296

[B76] Kamarajan P. Ateia I. Shin J. M. Fenno J. C. Le C. Zhan L.. (2020). Periodontal Pathogens Promote Cancer Aggressivity *via* TLR/MyD88 Triggered Activation of Integrin/FAK Signaling That Is Therapeutically Reversible by a Probiotic Bacteriocin. PloS Pathog. 16 (10), e1008881. doi: 10.1371/journal.ppat.1008881 33002094 PMC7529280

[B77] Kamarajan P. Hayami T. Matte B. Liu Y. Danciu T. Ramamoorthy A.. (2015). A Bacteriocin and Food Preservative, Inhibits Head and Neck Cancer Tumorigenesis and Prolongs Survival. PloS One 10 (7), e0131008. doi: 10.1371/journal.pone.0131008 26132406 PMC4489501

[B78] Kang W. Jia Z. Tang D. Zhang Z. Gao H. He K.. (2019). Fusobacterium Nucleatum Facilitates Apoptosis, ROS Generation, and Inflammatory Cytokine Production by Activating AKT/MAPK and NF-κb Signaling Pathways in Human Gingival Fibroblasts. Oxid. Med. Cell Longev. 2019, 1681972. doi: 10.1155/2019/1681972 31737164 PMC6815639

[B79] Kang W. Ji X. Zhang X. Tang D. Feng Q. (2019). Persistent Exposure to Fusobacterium Nucleatum Triggers Chemokine/Cytokine Release and Inhibits the Proliferation and Osteogenic Differentiation Capabilities of Human Gingiva-Derived Mesenchymal Stem Cells. Front. Cell Infect. Microbiol. 9, 429. doi: 10.3389/fcimb.2019.00429 31921705 PMC6927917

[B80] Kang J. H. Kim D. J. Choi B. K. Park J. W. (2017). Inhibition of Malodorous Gas Formation by Oral Bacteria With Cetylpyridinium and Zinc Chloride. Arch. Oral. Biol. 84, 133–138. doi: 10.1016/j.archoralbio.2017.09.023 28987726

[B81] Kang W. Sun T. Tang D. Zhou J. Feng Q. (2019). Time-Course Transcriptome Analysis of Gingiva-Derived Mesenchymal Stem Cells Reveals That Fusobacterium Nucleatum Triggers Oncogene Expression in the Process of Cell Differentiation. Front. Cell Dev. Biol. 7, 359. doi: 10.3389/fcell.2019.00359 31993418 PMC6970952

[B82] Kaplan A. Kaplan C. W. He X. McHardy I. Shi W. Lux R. (2014). Characterization of Aid1, a Novel Gene Involved in Fusobacterium Nucleatum Interspecies Interactions. Microb. Ecol. 68 (2), 379–387. doi: 10.1007/s00248-014-0400-y 24643713 PMC4104215

[B83] Kaplan C. W. Lux R. Haake S. K. Shi W. (2009). The Fusobacterium Nucleatum Outer Membrane Protein RadD Is an Arginine-Inhibitable Adhesin Required for Inter-Species Adherence and the Structured Architecture of Multispecies Biofilm. Mol. Microbiol. 71 (1), 35–47. doi: 10.1111/j.1365-2958.2008.06503.x 19007407 PMC2741168

[B84] Kawashima N. Suzuki N. Yang G. Ohi C. Okuhara S. Nakano-Kawanishi H.. (2007). Kinetics of RANKL, RANK and OPG Expressions in Experimentally Induced Rat Periapical Lesions. Oral. Surg. Oral. Med. Oral. Pathol. Oral. Radiol. Endod. 103 (5), 707–711. doi: 10.1016/j.tripleo.2006.11.036 17336108

[B85] Kenig A. Kessler A. Alaa S. Hazu W. Michael-Gaygo A. Amit S.. (2020). An Immunocompetent Patient With Culture-Negative Multiple Brain Abscesses Caused by Fusobacterium Nucleatum. Anaerobe 65, 102261. doi: 10.1016/j.anaerobe.2020.102261 32841677

[B86] Kim H. S. Lee D. S. Chang Y. H. Kim M. J. Koh S. Kim J.. (2010). Application of rpoB and Zinc Protease Gene for Use in Molecular Discrimination of Fusobacterium Nucleatum Subspecies. J. Clin. Microbiol. 48 (2), 545–553. doi: 10.1128/JCM.01631-09 19955278 PMC2815611

[B87] Kolenbrander P. E. (2000). Oral Microbial Communities: Biofilms, Interactions, and Genetic Systems. Annu. Rev. Microbiol. 54, 413–437. doi: 10.1146/annurev.micro.54.1.413 11018133

[B88] Komiya Y. Shimomura Y. Higurashi T. Sugi Y. Arimoto J. Umezawa S.. (2019). Patients With Colorectal Cancer Have Identical Strains of Fusobacterium Nucleatum in Their Colorectal Cancer and Oral Cavity. Gut 68 (7), 1335–1337. doi: 10.1136/gutjnl-2018-316661 29934439 PMC6582823

[B89] Kook J. K. Park S. N. Lim Y. K. Choi M. H. Cho E. Kong S. W.. (2013). Fusobacterium Nucleatum Subsp. Fusiforme Gharbia and Shah 1992 Is a Later Synonym of Fusobacterium Nucleatum Subsp. Vincentii Dzink Et al., 1990. Curr. Microbiol. 66 (4), 414–417. doi: 10.1007/s00284-012-0289-y 23263257

[B90] Kook J. K. Park S. N. Lim Y. K. Cho E. Jo E. Roh H.. (2017). Genome-Based Reclassification of Fusobacterium Nucleatum Subspecies at the Species Level. Curr. Microbiol. 74 (10), 1137–1147. doi: 10.1007/s00284-017-1296-9 28687946

[B91] Kostic A. D. Chun E. Robertson L. Glickman J. N. Gallini C. A. Michaud M.. (2013). Fusobacterium Nucleatum Potentiates Intestinal Tumorigenesis and Modulates the Tumor-Immune Microenvironment. Cell Host Microbe 14 (2), 207–215. doi: 10.1016/j.chom.2013.07.007 23954159 PMC3772512

[B92] Kostic A. D. Gevers D. Pedamallu C. S. Michaud M. Duke F. Earl A. M.. (2012). Genomic Analysis Identifies Association of Fusobacterium With Colorectal Carcinoma. Genome Res. 22 (2), 292–298. doi: 10.1101/gr.126573.111 22009990 PMC3266036

[B93] Krespi Y. P. Shrime M. G. Kacker A. (2006). The Relationship Between Oral Malodor and Volatile Sulfur Compound-Producing Bacteria. Otolaryngol. Head Neck Surg. 135 (5), 671–676. doi: 10.1016/j.otohns.2005.09.036 17071291

[B94] Kuper H. Adami H. O. Trichopoulos D. (2000). Infections as a Major Preventable Cause of Human Cancer. J. Intern. Med. 248 (3), 171–183. doi: 10.1046/j.1365-2796.2000.00742.x 10971784

[B95] Kurgan Ş. Kansal S. Nguyen D. Stephens D. Koroneos Y. Hasturk H.. (2017). Strain-Specific Impact of Fusobacterium Nucleatum on Neutrophil Function. J. Periodontol. 88 (4), 380–389. doi: 10.1902/jop.2016.160212 27762731

[B96] Lee C. H. Chang J. S. Syu S. H. Wong T. S. Chan J. Y. Tang Y. C.. (2015). IL-1β Promotes Malignant Transformation and Tumor Aggressiveness in Oral Cancer. J. Cell Physiol. 230 (4), 875–884. doi: 10.1002/jcp.24816 25204733

[B97] Lee M. J. Ha Y. E. Park H. Y. Lee J. H. Lee Y. J. Sung K. S.. (2012). Osteomyelitis of a Long Bone Due to Fusobacterium Nucleatum and Actinomyces Meyeri in an Immunocompetent Adult: A Case Report and Literature Review. BMC Infect. Dis. 12, 161. doi: 10.1186/1471-2334-12-161 22817336 PMC3481430

[B98] Lee P. Tan K. S. (2014). Fusobacterium Nucleatum Activates the Immune Response Through Retinoic Acid-Inducible Gene I. J. Dent. Res. 93 (2), 162–168. doi: 10.1177/0022034513516346 24334410

[B99] Lew H. P. Quah S. Y. Lui J. N. Bergenholtz G. Hoon Yu V. S. Tan K. S. (2015). Isolation of Alkaline-Tolerant Bacteria From Primary Infected Root Canals. J. Endod. 41 (4), 451–456. doi: 10.1016/j.joen.2014.12.003 25638530

[B100] Lima B. P. Shi W. Lux R. (2017). Identification and Characterization of a Novel Fusobacterium Nucleatum Adhesin Involved in Physical Interaction and Biofilm Formation With Streptococcus Gordonii. Microbiologyopen 6 (3), e00444. doi: 10.1002/mbo3.444 PMC5458471 28173636

[B101] Liu P. F. Haake S. K. Gallo R. L. Huang C. M. (2009). A Novel Vaccine Targeting Fusobacterium Nucleatum Against Abscesses and Halitosis. Vaccine 27 (10), 1589–1595. doi: 10.1016/j.vaccine.2008.12.058 19162109 PMC3057132

[B102] Liu J. Hsieh C. L. Gelincik O. Devolder B. Sei S. Zhang S.. (2019). Proteomic Characterization of Outer Membrane Vesicles From Gut Mucosa-Derived Fusobacterium Nucleatum. J. Proteomics 195, 125–137. doi: 10.1016/j.jprot.2018.12.029 30634002

[B103] Liu L. Liang L. Liang H. Wang M. Lu B. Xue M.. (2019). Fusobacterium Nucleatum Aggravates the Progression of Colitis by Regulating M1 Macrophage Polarization *via* AKT2 Pathway. Front. Immunol. 10, 1324. doi: 10.3389/fimmu.2019.01324 31249571 PMC6582778

[B104] Liu L. Liang L. Yang C. Zhou Y. Chen Y. (2021). Extracellular Vesicles of Fusobacterium Nucleatum Compromise Intestinal Barrier Through Targeting RIPK1-Mediated Cell Death Pathway. Gut Microbes 13 (1), 1–20. doi: 10.1080/19490976.2021.1902718 PMC8007154 33769187

[B105] Liu P. Liu Y. Wang J. Guo Y. Zhang Y. Xiao S. (2014). Detection of Fusobacterium Nucleatum and fadA Adhesin Gene in Patients With Orthodontic Gingivitis and Non-Orthodontic Periodontal Inflammation. PloS One 9 (1), e85280. doi: 10.1371/journal.pone.0085280 24416378 PMC3887018

[B106] Liu H. Redline R. W. Han Y. W. (2007). Fusobacterium Nucleatum Induces Fetal Death in Mice *via* Stimulation of TLR4-Mediated Placental Inflammatory Response. J. Immunol. 179 (4), 2501–2508. doi: 10.4049/jimmunol.179.4.2501 17675512

[B107] Liu P. F. Shi W. Zhu W. Smith J. W. Hsieh S. L. Gallo R. L.. (2010). Vaccination Targeting Surface FomA of Fusobacterium Nucleatum Against Bacterial Co-Aggregation: Implication for Treatment of Periodontal Infection and Halitosis. Vaccine 28 (19), 3496–3505. doi: 10.1016/j.vaccine.2010.02.047 20189489 PMC2855893

[B108] Llama-Palacios A. Potupa O. Sánchez M. C. Figuero E. Herrera D. Sanz M. (2020). Proteomic Analysis of Fusobacterium Nucleatum Growth in Biofilm Versus Planktonic State. Mol. Oral. Microbiol. 35 (4), 168–180. doi: 10.1111/omi.12303 32558324

[B109] Ma Z. Bi Q. Wang Y. (2015). Hydrogen Sulfide Accelerates Cell Cycle Progression in Oral Squamous Cell Carcinoma Cell Lines. Oral. Dis. 21 (2), 156–162. doi: 10.1111/odi.12223 24589248

[B110] Macia L. Nanan R. Hosseini-Beheshti E. Grau G. E. (2019). Host- and Microbiota-Derived Extracellular Vesicles, Immune Function, and Disease Development. Int. J. Mol. Sci. 21 (1), 107. doi: 10.3390/ijms21010107 PMC6982009 31877909

[B111] Maciel K. F. Neves de Brito L. C. Tavares W. L. Moreira G. Nicoli J. R. Vieira L. Q.. (2012). Cytokine Expression in Response to Root Canal Infection in Gnotobiotic Mice. Int. Endod. J. 45 (4), 354–362. doi: 10.1111/j.1365-2591.2011.01983.x 22233143

[B112] Malik A. Kanneganti T. D. (2017). Inflammasome Activation and Assembly at a Glance. J. Cell Sci. 130 (23), 3955–3963. doi: 10.1242/jcs.207365 29196474 PMC5769591

[B113] Martinho F. C. Leite F. R. Chiesa W. M. Nascimento G. G. Feres M. Gomes B. P. (2014). Signaling Pathways Activation by Primary Endodontic Infectious Contents and Production of Inflammatory Mediators. J. Endod. 40 (4), 484–489. doi: 10.1016/j.joen.2013.10.022 24666896

[B114] Meng Q. Gao Q. Mehrazarin S. Tangwanichgapong K. Wang Y. Huang Y.. (2021). Fusobacterium Nucleatum Secretes Amyloid-Like FadA to Enhance Pathogenicity. EMBO Rep. 22 (7), e52891. doi: 10.15252/embr.202152891 34184813 PMC8406402

[B115] Mitsuhashi K. Nosho K. Sukawa Y. Matsunaga Y. Ito M. Kurihara H.. (2015). Association of Fusobacterium Species in Pancreatic Cancer Tissues With Molecular Features and Prognosis. Oncotarget 6 (9), 7209–7220. doi: 10.18632/oncotarget.3109 25797243 PMC4466679

[B116] Mittal V. (2018). Epithelial Mesenchymal Transition in Tumor Metastasis. Annu. Rev. Pathol. 13, 395–412. doi: 10.1146/annurev-pathol-020117-043854 29414248

[B117] Nagy K. N. Sonkodi I. Szöke I. Nagy E. Newman H. N. (1998). The Microflora Associated With Human Oral Carcinomas. Oral. Oncol. 34 (4), 304–308. doi: 10.1016/S1368-8375(98)80012-2 9813727

[B118] Nani B. D. Lima P. O. Marcondes F. K. Groppo F. C. Rolim G. S. Moraes A. B.. (2017). Changes in Salivary Microbiota Increase Volatile Sulfur Compounds Production in Healthy Male Subjects With Academic-Related Chronic Stress. PloS One 12 (3), e0173686. doi: 10.1371/journal.pone.0173686 28319129 PMC5358872

[B119] Parhi L. Alon-Maimon T. Sol A. Nejman D. Shhadeh A. Fainsod-Levi T.. (2020). Breast Cancer Colonization by Fusobacterium Nucleatum Accelerates Tumor Growth and Metastatic Progression. Nat. Commun. 11 (1), 3259. doi: 10.1038/s41467-020-16967-2 32591509 PMC7320135

[B120] Patini R. Staderini E. Lajolo C. Lopetuso L. Mohammed H. Rimondini L.. (2018). Relationship Between Oral Microbiota and Periodontal Disease: A Systematic Review. Eur. Rev. Med. Pharmacol. Sci. 22 (18), 5775–5788. doi: 10.26355/eurrev_201809_15903 30280756

[B121] Pereira R. S. Rodrigues V. A. A. Furtado W. T. Gueiros S. Pereira G. S. Avila-Campos M. J. (2017). Microbial Analysis of Root Canal and Periradicular Lesion Associated to Teeth With Endodontic Failure. Anaerobe 48, 12–18. doi: 10.1016/j.anaerobe.2017.06.016 28666877

[B122] Polak D. Wilensky A. Shapira L. Halabi A. Goldstein D. Weiss E. I.. (2009). Mouse Model of Experimental Periodontitis Induced by Porphyromonas Gingivalis/Fusobacterium Nucleatum Infection: Bone Loss and Host Response. J. Clin. Periodontol. 36 (5), 406–410. doi: 10.1111/j.1600-051X.2009.01393.x 19419440

[B123] Rai M. Spratt D. Gomez-Pereira P. R. Patel J. Nair S. P. (2016). Light Activated Antimicrobial Agents can Inactivate Oral Malodour Causing Bacteria. J. Breath Res. 10 (4), 046009. doi: 10.1088/1752-7155/10/4/046009 27753430

[B124] Rôças I. N. Siqueira J. F. Jr. (2012). Characterization of Microbiota of Root Canal-Treated Teeth With Posttreatment Disease. J. Clin. Microbiol. 50 (5), 1721–1724. doi: 10.1128/JCM.00531-12 22403423 PMC3347098

[B125] Rodríguez Duque J. C. Galindo Rubín P. González Humara B. Quesada Sanz A. A. Busta Vallina M. B. Fernández-Sampedro M. (2018). Fusobacterium Nucleatum Prosthetic Hip Infection: Case Report and Review of the Literature of Unusual Anaerobic Prosthetic Joint Infection. Anaerobe 54, 75–82. doi: 10.1016/j.anaerobe.2018.08.003 30118892

[B126] Rubinstein M. R. Wang X. Liu W. Hao Y. Cai G. Han Y. W. (2013). Fusobacterium Nucleatum Promotes Colorectal Carcinogenesis by Modulating E-Cadherin/β-Catenin Signaling *via* its FadA Adhesin. Cell Host Microbe 14 (2), 195–206. doi: 10.1016/j.chom.2013.07.012 23954158 PMC3770529

[B127] Rui M. Zhang X. Huang J. Wei D. Li Z. Shao Z.. (2021). The Baseline Oral Microbiota Predicts the Response of Locally Advanced Oral Squamous Cell Carcinoma Patients to Induction Chemotherapy: A Prospective Longitudinal Study. Radiother. Oncol. 164, 83–91. doi: 10.1016/j.radonc.2021.09.013 34571091

[B128] Saito A. Kokubu E. Inagaki S. Imamura K. Kita D. Lamont R. J.. (2012). Porphyromonas Gingivalis Entry Into Gingival Epithelial Cells Modulated by Fusobacterium Nucleatum is Dependent on Lipid Rafts. Microb. Pathog. 53 (5-6), 234–242. doi: 10.1016/j.micpath.2012.08.005 23034475 PMC3653298

[B129] Sassone L. M. Fidel R. A. Faveri M. Guerra R. Figueiredo L. Fidel S. R.. (2008). A Microbiological Profile of Symptomatic Teeth With Primary Endodontic Infections. J. Endod. 34 (5), 541–545. doi: 10.1016/j.joen.2008.02.004 18436031

[B130] Senkus K. E. Crowe-White K. M. (2020). Influence of Mouth Rinse Use on the Enterosalivary Pathway and Blood Pressure Regulation: A Systematic Review. Crit. Rev. Food Sci. Nutr. 60 (17), 2874–2886. doi: 10.1080/10408398.2019.1665495 31542940

[B131] Settem R. P. El-Hassan A. T. Honma K. Stafford G. P. Sharma A. (2012). Fusobacterium Nucleatum and Tannerella Forsythia Induce Synergistic Alveolar Bone Loss in a Mouse Periodontitis Model. Infect. Immun. 80 (7), 2436–2443. doi: 10.1128/IAI.06276-11 22547549 PMC3416462

[B132] Shinada K. Ueno M. Konishi C. Takehara S. Yokoyama S. Zaitsu T.. (2010). Effects of a Mouthwash With Chlorine Dioxide on Oral Malodor and Salivary Bacteria: A Randomized Placebo-Controlled 7-Day Trial. Trials 11, 14. doi: 10.1186/1745-6215-11-14 20152022 PMC2831889

[B133] Sigusch B. W. Engelbrecht M. Völpel A. Holletschke A. Pfister W. Schütze J. (2010). Full-Mouth Antimicrobial Photodynamic Therapy in Fusobacterium Nucleatum-Infected Periodontitis Patients. J. Periodontol. 81 (7), 975–981. doi: 10.1902/jop.2010.090246 20350153

[B134] Su S. C. Chang L. C. Huang H. D. Peng C. Y. Chuang C. Y. Chen Y. T.. (2021). Oral Microbial Dysbiosis and its Performance in Predicting Oral Cancer. Carcinogenesis 42 (1), 127–135. doi: 10.1093/carcin/bgaa062 32621740

[B135] Sun X. Yang X. Xue P. Zhang Z. Ren G. (2019). Improved Antibacterial Effects of Alkali-Transformed Saponin From Quinoa Husks Against Halitosis-Related Bacteria. BMC Complement. Altern. Med. 19 (1), 46. doi: 10.1186/s12906-019-2455-2 30755185 PMC6373059

[B136] Suzuki N. Nakano Y. Watanabe T. Yoneda M. Hirofuji T. Hanioka T. (2018). Two Mechanisms of Oral Malodor Inhibition by Zinc Ions. J. Appl. Oral. Sci. 26, e20170161. doi: 10.1590/1678-7757-2017-0161 29364345 PMC5777415

[B137] Suzuki N. Yoneda M. Tanabe K. Fujimoto A. Iha K. Seno K.. (2014). Lactobacillus Salivarius WB21–containing Tablets for the Treatment of Oral Malodor: A Double-Blind, Randomized, Placebo-Controlled Crossover Trial. Oral. Surg. Oral. Med. Oral. Pathol. Oral. Radiol. 117 (4), 462–470. doi: 10.1016/j.oooo.2013.12.400 24556493

[B138] Swaminathan N. Aguilar F. (2020). Cryptogenic Pyogenic Liver Abscess Due to Fusobacterium Nucleatum in an Immunocompetent Patient. Eur. J. Case Rep. Intern. Med. 7 (10), 001741. doi: 10.12890/2020_001741 33083352 PMC7546556

[B139] Swidsinski A. Dörffel Y. Loening-Baucke V. Theissig F. Rückert J. C. Ismail M.. (2011). Acute Appendicitis Is Characterised by Local Invasion With Fusobacterium Nucleatum/Necrophorum. Gut 60 (1), 34–40. doi: 10.1136/gut.2009.191320 19926616

[B140] Taxman D. J. Swanson K. V. Broglie P. M. Wen H. Holley-Guthrie E. Huang M. T.. (2012). Porphyromonas Gingivalis Mediates Inflammasome Repression in Polymicrobial Cultures Through a Novel Mechanism Involving Reduced Endocytosis. J. Biol. Chem. 287 (39), 32791–32799. doi: 10.1074/jbc.M112.401737 22843689 PMC3463344

[B141] Teixeira-Salum T. B. Rodrigues D. B. Gervásio A. M. Souza C. J. Rodrigues V. Jr. Loyola A. M. (2010). Distinct Th1, Th2 and Treg Cytokines Balance in Chronic Periapical Granulomas and Radicular Cysts. J. Oral. Pathol. Med. 39 (3), 250–256. doi: 10.1111/j.1600-0714.2009.00863.x 20102461

[B142] Témoin S. Wu K. L. Wu V. Shoham M. Han Y. W. (2012). Signal Peptide of FadA Adhesin From Fusobacterium Nucleatum Plays a Novel Structural Role by Modulating the Filament’s Length and Width. FEBS Lett. 586 (1), 1–6. doi: 10.1016/j.febslet.2011.10.047 22108653 PMC3249520

[B143] Tezal M. Sullivan M. A. Hyland A. Marshall J. R. Stoler D. Reid M. E.. (2009). Chronic Periodontitis and the Incidence of Head and Neck Squamous Cell Carcinoma. Cancer Epidemiol. Biomarkers Prev. 18 (9), 2406–2412. doi: 10.1158/1055-9965.EPI-09-0334 19745222

[B144] Truant A. L. Menge S. Milliorn K. Lairscey R. Kelly M. T. (1983). Fusobacterium Nucleatum Pericarditis. J. Clin. Microbiol. 17 (2), 349–351. doi: 10.1128/jcm.17.2.349-351.1983 6833485 PMC272635

[B145] Uitto V. J. Baillie D. Wu Q. Gendron R. Grenier D. Putnins E. E.. (2005). Fusobacterium Nucleatum Increases Collagenase 3 Production and Migration of Epithelial Cells. Infect. Immun. 73 (2), 1171–1179. doi: 10.1128/IAI.73.2.1171-1179.2005 15664960 PMC547012

[B146] Umaña A. Sanders B. E. Yoo C. C. Casasanta M. A. Udayasuryan B. Verbridge S. S.. (2019). Utilizing Whole Fusobacterium Genomes To Identify, Correct, and Characterize Potential Virulence Protein Families. J. Bacteriol. 201 (23), e00273-19. doi: 10.1128/JB.00273-19 31501282 PMC6832068

[B147] Vander Haar E. L. So J. Gyamfi-Bannerman C. Han Y. W. (2018). Fusobacterium Nucleatum and Adverse Pregnancy Outcomes: Epidemiological and Mechanistic Evidence. Anaerobe 50, 55–59. doi: 10.1016/j.anaerobe.2018.01.008 29409815 PMC6750227

[B148] Vital M. Howe A. C. Tiedje J. M. (2014). Revealing the Bacterial Butyrate Synthesis Pathways by Analyzing (Meta)Genomic Data. mBio 5 (2), e00889. doi: 10.1128/mBio.00889-14 24757212 PMC3994512

[B149] Wang X. Buhimschi C. S. Temoin S. Bhandari V. Han Y. W. Buhimschi I. A. (2013). Comparative Microbial Analysis of Paired Amniotic Fluid and Cord Blood From Pregnancies Complicated by Preterm Birth and Early-Onset Neonatal Sepsis. PloS One 8 (2), e56131. doi: 10.1371/journal.pone.0056131 23437088 PMC3577789

[B150] Wang P. Duan D. Zhou X. Li X. Yang J. Deng M.. (2015). Relationship Between Expression of Human Gingival Beta-Defensins and Levels of Periodontopathogens in Subgingival Plaque. J. Periodontal. Res. 50 (1), 113–122. doi: 10.1111/jre.12187 24814979

[B151] Wang F. Qiao W. Bao B. Wang S. Regenstein J. Shi Y.. (2019). Effect of IgY on Periodontitis and Halitosis Induced by Fusobacterium Nucleatum. J. Microbiol. Biotechnol. 29 (2), 311–320. doi: 10.4014/jmb.1810.10044 30609885

[B152] Wu J. Li Q. Fu X. (2019). Fusobacterium Nucleatum Contributes to the Carcinogenesis of Colorectal Cancer by Inducing Inflammation and Suppressing Host Immunity. Transl. Oncol. 12 (6), 846–851. doi: 10.1016/j.tranon.2019.03.003 30986689 PMC6462820

[B153] Xue P. Yao Y. Yang X. S. Feng J. Ren G. X. (2017). Improved Antimicrobial Effect of Ginseng Extract by Heat Transformation. J. Ginseng Res. 41 (2), 180–187. doi: 10.1016/j.jgr.2016.03.002 28413322 PMC5386132

[B154] Xu M. Yamada M. Li M. Liu H. Chen S. G. Han Y. W. (2007). FadA From Fusobacterium Nucleatum Utilizes Both Secreted and Nonsecreted Forms for Functional Oligomerization for Attachment and Invasion of Host Cells. J. Biol. Chem. 282 (34), 25000–25009. doi: 10.1074/jbc.M611567200 17588948

[B155] Yamamura K. Baba Y. Nakagawa S. Mima K. Miyake K. Nakamura K.. (2016). Human Microbiome Fusobacterium Nucleatum in Esophageal Cancer Tissue Is Associated With Prognosis. Clin. Cancer Res. 22 (22), 5574–5581. doi: 10.1158/1078-0432.CCR-16-1786 27769987

[B156] Yamamura K. Izumi D. Kandimalla R. Sonohara F. Baba Y. Yoshida N.. (2019). Intratumoral Fusobacterium Nucleatum Levels Predict Therapeutic Response to Neoadjuvant Chemotherapy in Esophageal Squamous Cell Carcinoma. Clin. Cancer Res. 25 (20), 6170–6179. doi: 10.1158/1078-0432.CCR-19-0318 31358543 PMC6801075

[B157] Yang Y. Weng W. Peng J. Hong L. Yang L. Toiyama Y.. (2017). Fusobacterium Nucleatum Increases Proliferation of Colorectal Cancer Cells and Tumor Development in Mice by Activating Toll-Like Receptor 4 Signaling to Nuclear Factor-κb, and Up-Regulating Expression of MicroRNA-21. Gastroenterology 152 (4), 851–866.e24. doi: 10.1053/j.gastro.2016.11.018 27876571 PMC5555435

[B158] Yang C. Y. Yeh Y. M. Yu H. Y. Chin C. Y. Hsu C. W. Liu H.. (2018). Oral Microbiota Community Dynamics Associated With Oral Squamous Cell Carcinoma Staging. Front. Microbiol. 9, 862. doi: 10.3389/fmicb.2018.00862 29774014 PMC5943489

[B159] Yost S. Stashenko P. Choi Y. Kukuruzinska M. Genco C. A. Salama A.. (2018). Increased Virulence of the Oral Microbiome in Oral Squamous Cell Carcinoma Revealed by Metatranscriptome Analyses. Int. J. Oral. Sci. 10 (4), 32. doi: 10.1038/s41368-018-0037-7 30420594 PMC6232154

[B160] Yu T. Guo F. Yu Y. Sun T. Ma D. Han J.. (2017). Fusobacterium Nucleatum Promotes Chemoresistance to Colorectal Cancer by Modulating Autophagy. Cell 170 (3), 548–563.e16. doi: 10.1016/j.cell.2017.07.008 28753429 PMC5767127

[B161] Zhang S. Bian H. Li X. Wu H. Bi Q. Yan Y.. (2016). Hydrogen Sulfide Promotes Cell Proliferation of Oral Cancer Through Activation of the COX2/AKT/ERK1/2 Axis. Oncol. Rep. 35 (5), 2825–2832. doi: 10.3892/or.2016.4691 26987083

[B162] Zhang Z. Liu S. Zhang S. Li Y. Shi X. Liu D. (2021). Porphyromonas Gingivalis Outer Membrane Vesicles Inhibit the Invasion of Fusobacterium Nucleatum Into Oral Epithelial Cells by Downregulating FadA and FomA. J. Periodontol. 2021. doi: 10.1002/JPER.21-0144 PMC9415117 34458990

[B163] Zhang L. Liu Y. Zheng H. J. Zhang C. P. (2019). The Oral Microbiota May Have Influence on Oral Cancer. Front. Cell Infect. Microbiol. 9, 476. doi: 10.3389/fcimb.2019.00476 32010645 PMC6974454

[B164] Zhang S. Li C. Zhang Z. Li Y. Li Q. Geng F.. (2021). Analysis of Differentially Expressed Genes in Oral Epithelial Cells Infected With Fusobacterium Nucleatum for Revealing Genes Associated With Oral Cancer. J. Cell Mol. Med. 25 (2), 892–904. doi: 10.1111/jcmm.16142 33289330 PMC7812288

[B165] Zheng D. W. Dong X. Pan P. Chen K. W. Fan J. X. Cheng S. X.. (2019). Phage-Guided Modulation of the Gut Microbiota of Mouse Models of Colorectal Cancer Augments Their Responses to Chemotherapy. Nat. BioMed. Eng. 3 (9), 717–728. doi: 10.1038/s41551-019-0423-2 31332342

[B166] Zupan J. Jeras M. Marc J. (2013). Osteoimmunology and the Influence of Pro-Inflammatory Cytokines on Osteoclasts. Biochem. Med. (Zagreb) 23 (1), 43–63. doi: 10.11613/BM.2013.007 23457765 PMC3900089

